# Protein Lactylation and Metabolic Regulation of the Zoonotic Parasite *Toxoplasma gondii*

**DOI:** 10.1016/j.gpb.2022.09.010

**Published:** 2022-10-07

**Authors:** Deqi Yin, Ning Jiang, Chang Cheng, Xiaoyu Sang, Ying Feng, Ran Chen, Qijun Chen

**Affiliations:** 1 Key Laboratory of Livestock Infectious Diseases in Northeast China, Ministry of Education, Key Laboratory of Zoonosis, College of Animal Science and Veterinary Medicine, Shenyang Agricultural University, Shenyang 110166, China; 2 The Research Unit for Pathogenic Mechanisms of Zoonotic Parasites, Chinese Academy of Medical Sciences, Shenyang 110866, China

**Keywords:** *Toxoplasma gondii*, Lactylation, Protein post-translational modification, Metabolism, ChIP-seq

## Abstract

The biology of ***Toxoplasma gondii***, the causative pathogen of one of the most widespread parasitic diseases (toxoplasmosis), remains poorly understood. Lactate, which is derived from glucose **metabolism**, is not only an energy source in a variety of organisms**,** including *T*. *gondii*, but also a regulatory molecule that participates in gene activation and protein function. Lysine **lactylation** (Kla) is a type of post-translational modifications (PTMs) that has been recently associated with chromatin remodeling; however, Kla of histone and non-histone proteins has not yet been studied in *T*. *gondii*. To examine the prevalence and function of lactylation in *T*. *gondii* parasites, we mapped the lactylome of proliferating tachyzoite cells and identified 1964 Kla sites on 955 proteins in the *T*. *gondii* RH strain. Lactylated proteins were distributed in multiple subcellular compartments and were closely related to a wide variety of biological processes, including mRNA splicing, glycolysis, aminoacyl-tRNA biosynthesis, RNA transport**,** and many signaling pathways. We also performed a chromatin immunoprecipitation sequencing (**ChIP-seq**) analysis using a lactylation-specific antibody and found that the histones H4K12la and H3K14la were enriched in the promoter and exon regions of *T*. *gondii* associated with microtubule-based movement and cell invasion. We further confirmed the delactylase activity of histone deacetylases TgHDAC2–4, and found that treatment with anti-histone acetyltransferase (TgMYST-A) antibodies profoundly reduced protein lactylation in *T*. *gondii*. This study offers the first dataset of the global lactylation proteome and provides a basis for further dissecting the functional biology of *T*. *gondii*.

## Introduction

*Toxoplasma gondii* is an exclusively intracellular apicomplexan parasite that infects all nucleated vertebrate cells, and a globally prevalent protozoan parasite that infects more than 30% of the world population [1]. This parasite is a major opportunistic pathogen that causes toxoplasmosis, which is considered one of the most widespread zoonotic diseases [2]. *T*. *gondii* has a complicated life cycle, which mainly involves sexual and asexual phases, and primarily exists in two infectious forms in the tissues of its intermediate hosts (warm-blooded animals): rapidly replicating tachyzoites and slowly dividing bradyzoites [3]. Although the parasite typically causes mild symptoms in immunocompetent individuals, developing fetuses and immunocompromised individuals develop severe symptoms that might even be life-threatening [4].

After invading a host cell, *T*. *gondii* disseminates in the form of rapidly replicating haploid tachyzoites throughout nucleated cells in the host. In certain organs and tissue types, tachyzoites transform into bradyzoites to form cysts (semi-dormant form associated with slow replication), thereby establishing a continuous latent infection in the host. Once the host’s immune system is weakened, bradyzoites are activated and transformed into tachyzoites to reinfect cells [5]. Sexual reproduction of *T*. *gondii* occurs in the intestinal epithelial cells of felines (the terminal hosts), and parasites in the form of oocysts are released with feces, thereby contaminating the environment. Humans and warm-blooded animals are typically infected through exposure to tissue cysts or oocyst-contaminated food or water. Currently, effective anti-*T*. *gondii* infection agents are restricted to some common metabolic inhibitors, including azithromycin, pyrimethamine, spiramycin, sulfonamides, trimethoprim–butoxypyrimidine, and clindamycin [6]. Notably, these agents share some common limitations, which includes being only effective on tachyzoites in the replication phase, exerting only a slight or even no effect on semi-dormant bradyzoites, and in some cases, causing severe cytotoxicity to the host. Therefore, further knowledge regarding the biological characteristics of *T*. *gondii* is warranted to facilitate the discovery of new drug targets.

Type I *T*. *gondii* strains (such as the RH strain) are highly virulent and lethal in mice, resulting in severe clinical manifestations. However, type II strains (such as the ME49 strain) exhibit relatively low pathogenicity in murine hosts, typically manifesting as chronic infections [7]. The proliferation of *T*. *gondii* is strictly dependent on precise gene expression control, which ensures appropriate protein profiles [4]. Post-translational modifications (PTMs) constitute one of the critical response mechanisms of *T*. *gondii* to external environmental stimulation and may control its lifecycle [8]. PTMs, which involve the addition of chemical moieties to one or more amino acid residues for the regulation of protein function, affect almost all aspects of *T*. *gondii* pathogenesis and cell biology [5]. Specifically, these modifications affect protein–protein interactions (PPIs), spatial conformation, subcellular localization, and stability of proteins by altering the physicochemical properties of proteins, and these effects significantly regulate the complex life cycle of the parasites. Therefore, revealing the regulatory mechanisms of PTMs in *T*. *gondii* will promote the discovery of parasite-specific methods for toxoplasmosis treatment and prevention. To date, more than 12 types of PTMs, namely, 2-hydroxyisobutyrylation, malonylation, acetylation, succinylation, crotonylation, ubiquitination, methylation, palmitoylation, N-myristoylation, SUMOylation, phosphorylation, and O-GlcNAcylation, have been discovered in *T*. *gondii* [9], [10], [11], [12], [13], [14], [15]. Among these PTMs, phosphorylation, which is a highly dynamic process that plays a vital role in cell signal transduction, represents the most common PTM in *T*. *gondii* [16]. We previously revealed that 2-hydroxyisobutyrylation covers approximately 23.2% and 20.5% of *T*. *gondii* RH and ME49 proteomes, respectively, and plays an essential role in gene expression, energy metabolism, and invasion [15]. In addition, 1061 crotonylated proteins were detected in the *T*. *gondii* RH strain and 984 in the ME49 strain, which are strictly related to energy metabolism, invasion, and protein synthesis and degradation [15]. Lysine acetylation, which represents approximately 5% of the predicted proteome of *T*. *gondii*, is associated with protein translation and metabolism [17], [18]. Ubiquitination accounts for up to 5% of the *T*. *gondii* proteome and is considered a key regulator of cell cycle transition [19]. Furthermore, 370 arginine monomethylated proteins, which cover approximately 4.5% of the proteome, are involved in transcriptional regulation, DNA repair, and RNA metabolism [10]. Collectively, PTMs are highly abundant in *T*. *gondii* and play integral roles in multiple vital biological pathways.

Lactate was originally thought to be a metabolite of glycolysis and an energy source [20], [21]. Lysine lactylation (Kla), a novel histone marker associated with gene activation, can be obtained from lactate (a cellular metabolite) and detected through a +86.03 Da mass shift of lysine residues [22]. Lactate is currently considered to be a precursor that stimulates lactylation and is produced via the glycolytic pathway.

In this study, we leveraged the first comprehensive analysis of the lactylome of *T*. *gondii* and dissected the regulatory potential of Kla, which opened a new avenue for elucidating parasite biology and identifying novel parasiticide targets.

## Results

### Identification of Kla in the proteome of *T*. *gondii* RH

**Figure 1:**
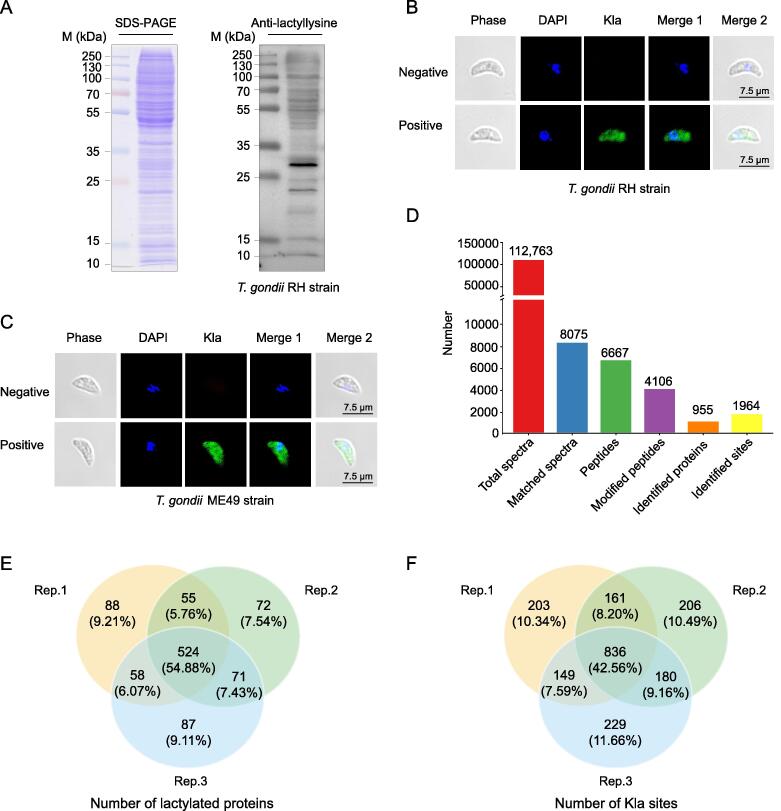
**Lactylation is widespread in*T. gondii*illustrated by Western blotting, IFA, and LC–MS/MS** **A.** SDS–PAGE and Western blotting analysis of tachyzoite lysate (20 μg) probed with an anti-lactyllysine antibody. **B.** IFA of tachyzoites of *T*. *gondii* RH with an anti-lactyllysine antibody (green). The negative control antibody is a normal rabbit IgG. Nuclei were stained with DAPI (blue). **C.** IFA of tachyzoites of *T*. *gondii* ME49 with an anti-lactyllysine antibody (green). The negative control antibody is a normal rabbit IgG. Nuclei were stained with DAPI (blue). **D.** LC–MS/MS data of lysine lactylation. **E.** Venn diagram showing the overlapping lactylated proteins obtained from triplicates. **F.** Venn diagram showing the overlapping Kla sites obtained from triplicate replications. M, marker; Kla, lysine lactylation; LC–MS/MS, liquid chromatography–tandem mass spectrometry; IFA, immunofluorescence assay; *T. gondii*, *Toxoplasma gondii*; SDS–PAGE, sodium dodecyl sulfate–polyacrylamide gel electrophoresis; IgG, immunoglobulin G; DAPI, 4′,6-diamidino-2-phenylindole.

To characterize the global Kla profile of *T*. *gondii*, whole tachyzoite lysates were analyzed using liquid chromatography–tandem mass spectrometry (LC–MS/MS) (Figure S1A). Protein lactylation in tachyzoites was verified through Western blotting using a pan anti-lactyllysine antibody. Multiple proteins were identified (Figure 1A) using this antibody. The presence of lactylated proteins in the tachyzoites of *T*. *gondii* RH and ME49 was also confirmed using an immunofluorescence assay (IFA) with an anti-L-lactyllysine antibody (Figure 1B and C). The results showed that lactylated proteins were widely distributed in multiple discrete compartments of *T*. *gondii*.

We identified 1964 Kla sites on 955 proteins in triplicate experiments (Figure 1D–F, Table S1). In addition, we evaluated the mass error to verify the accuracy of the MS data. The MaxQuant data were filtered according to the following thresholds: mass error ≤ 5 ppm and localization probability > 75%. The results suggested that mass validation of the large-scale data conform to the experimental standards (Figure S1B). In addition, the length distribution of the identified lactylated peptides met quality control requirements (Figure S1C). Most lactylated proteins (86.1%) contained 1–3 Kla sites, whereas approximately 5.4% of the proteins contained more than 5 Kla sites (Figure S1D). We identified three proteins containing the highest number of Kla sites: the translation elongation factor 2 family protein (16 sites), elongation factor 1-alpha (14 sites), and heat shock 70 protein (14 sites).

### Lactylated proteins are widely distributed in multiple subcellular compartments in *T*. *gondii*

To further explore the possible biological functions of the lactylated proteins, these proteins were statistically classified into Gene Ontology (GO) secondary annotations, as shown in Figure S2 and Table S2. The lactylated proteins were mainly distributed in intracellular organelles and were related to various molecular functions, such as protein binding, organic cyclic, and heterocyclic compound binding. The top three biological processes were cellular metabolic process, organic substance metabolic process, and primary metabolic process.

**Figure 2:**
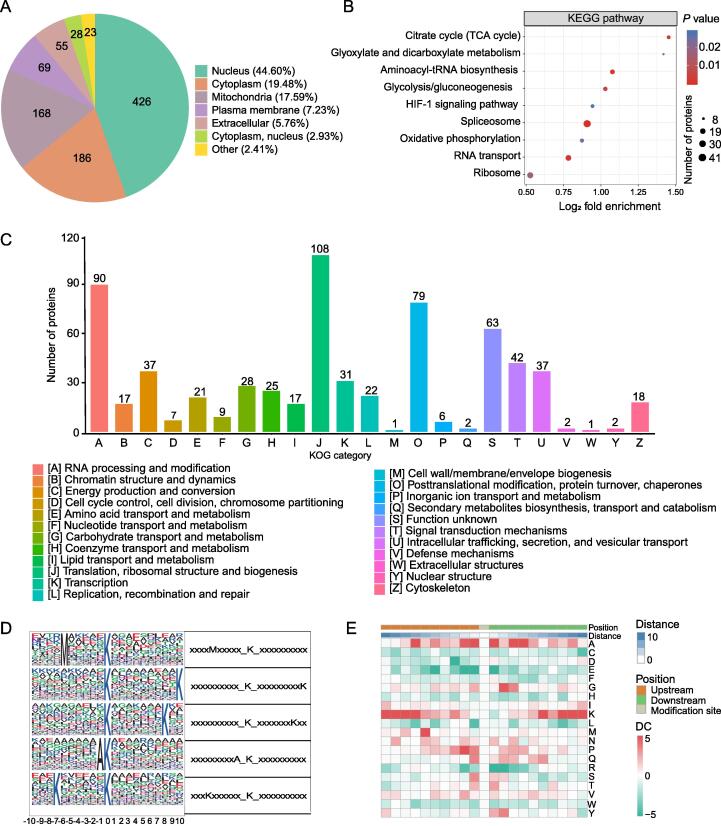
**Lactylated proteins are distributed in multiple subcellular compartments and involved in various biological functions** **A.** Subcellular distribution of the identified lactylated proteins. **B.** KEGG enrichment analysis of the lactylated proteins (Fisher’s exact test, *P* < 0.05). **C.** Functional classification of lactylated proteins based on the KOG database. **D.** Probability sequence motifs of lactylation sites consisting of 20 residues surrounding the targeted lysine residue were produced using Motif-x. The size of each letter corresponds to the frequency of that amino acid residue occurring in that position. **E.** A heatmap shows the high (red) or low (green) occurrence frequency of the amino acids surrounding the lactylated lysines. The red box indicates the amino acid occurring more frequently in that position, while the green box indicates the amino acid occurring less frequently  in that position. KEGG, Kyoto Encyclopedia of Genes and Genomes; DC, difference score; KOG, EuKaryotic Orthologous Groups.

Based on the annotation results, we performed a subcellular localization analysis of the lactylated proteins, as shown in Figure 2A and Table S3. The top three localizations of the identified proteins were in the nucleus (*n* = 426, 44.60%), cytoplasm (*n* = 186, 19.48%), and mitochondria (*n* = 168, 17.59%). A total of 69 (7.23%) and 55 (5.76%) proteins were distributed in the plasma membrane and the extracellular space, respectively.

### Lactylated proteins are enriched in multiple metabolic processes

The GO biological process category demonstrated that the lactylated proteins were strongly enriched in ribonucleotide monophosphate and triphosphate biosynthetic processes (Figure S3; Table S4). Furthermore, we constructed a GO-enriched directed acyclic graph (DAG) of each GO category to provide visual features of the lactylated proteins (Figure S4). Notably, mitochondrial components were extremely enriched in the DAG.

Protein domain enrichment revealed that the RNA recognition motif was the most highly enriched domain, implying a positive role for Kla in RNA-binding proteins (RBPs). Furthermore, the elongation factor Tu domain, pyridine nucleotide–disulfide oxidoreductase, ATP synthase alpha/beta family nucleotide-binding domain, and ATP synthase alpha/beta family beta-barrel domain were also predicted to be significantly enriched **(**Figure S5; Table S5).

As revealed by Kyoto Encyclopedia of Genes and Genomes (KEGG) pathway enrichment, lactylated proteins were mainly enriched in multiple central pathways, including the spliceosome, aminoacyl-tRNA biosynthesis, ribosome, RNA transport, glycolysis/gluconeogenesis, hypoxia-inducible factor signaling pathway, oxidative phosphorylation, glyoxylate and dicarboxylate metabolism, and tricarboxylic acid cycle (TCA cycle) (Figure 2B; Table S6).

The overall EuKaryotic Orthologous Group (KOG) tool has been developed as a tool for large-scale functional and evolutionary analyses of proteins. In this study, we performed a functional classification analysis of lactylated proteins based on the KOG database. The top five KOG terms obtained from the analysis of lactylated proteins were energy production and conversion [C]; signal transduction mechanisms [T]; RNA processing and modification [A]; translation, ribosomal structure and biogenesis [J]; and post-translational modification, protein turnover, and chaperones [O] (Figure 2C; Table S7).

### High-frequency lactylation in peptide regions with conserved motifs

The frequencies of lactylated amino acids (from −10 to +10) in all identified peptides were statistically analyzed using the MoMo software to assess the distinctive characteristics of the sequences surrounding the Kla sites. We retrieved five conserved motifs according to the enrichment statistics (Figure 2D**)**. Notably, the M-Kla, A-Kla, and K-Kla motifs were conserved. We established an enrichment heatmap to further analyze the motif sequences (Figure 2E; Table S8). The results showed that methionine (M), alanine (A), and lysine (K) residues often appeared upstream of the Kla sites and alanine (A), glycine (G), and lysine (K) residues were more frequently found downstream of the Kla sites.

### Lactylated proteins are involved in gene transcription and expression

The spliceosome machinery, which is widespread in eukaryotes, is mainly composed of five ribonucleoproteins, namely U1, U2, U4, U5, and U6 small nuclear RNA (snRNA), and mainly functions to remove transcript regions generated from introns [23]. In this study, 49 lactylated proteins were found to be closely associated with spliceosome (Figure S6; Table S9). Notably, 14.29% (7/49) of the proteins contained at least three Kla sites, including the DEAD/DEAH box helicase domain-containing protein, small nuclear ribonucleoprotein G, SKIP/SNW domain-containing protein, crooked neck family 1 protein isoform 2, RNA recognition motif-containing protein, pre-mRNA processing splicing factor PRP8, and heat shock protein HSP70. HSP70 may also be involved in splicing as a chaperone [24]. Strikingly, HSP70 was the protein with the highest number of Kla sites (*n* = 14), suggesting that it might play an essential role in the spliceosome of *T*. *gondii*.

### Enzymes involved in glycolysis/gluconeogenesis, the TCA cycle, and oxidative phosphorylation are predominantly lactylated

Glycolysis and the TCA cycle have been recognized as the main central carbon metabolic pathways of fast-growing tachyzoites and slowly developing bradyzoites in *T*. *gondii* [25], [26]. Here, the Kla of key enzymes that mediate glycolysis/gluconeogenesis and TCA cycle metabolic pathways were thoroughly analyzed (Figure S7). A total of 14 regulatory enzymes with 43 Kla sites were identified to be related to glycolysis/gluconeogenesis pathways (Table S10), including phosphoglycerate kinase (PGKI), fructose-1,6-bisphosphate aldolase (ALD2), glyceraldehyde-3-phosphate dehydrogenase (GAPDH1), fructose-1,6-bisphosphate aldolase (ALD1), glucosephosphate-mutase (GPM1), fructose-bisphosphate I (FBP 1), fructose-bisphosphate II (FBP 2), enolase 2 (ENO 2), enolase 1 (ENO 1), phosphoglycerate mutase PGMII, pyruvate kinase I (PyKI), pyruvate kinase II (PyKII), lactate dehydrogenase (LDH1), and hexokinase (HK). Notably, PGKI and ALD1 contained the most Kla sites (*n* = 6).

In the TCA cycle, 12 enzymes with 47 Kla sites were identified (Table S10), including citrate synthase I (CSI), aconitate hydratase ACN/IRP, isocitrate dehydrogenase (IDH), 2-oxoglutarate dehydrogenase e1 component, putative (2-ODE1), dihydrolipoyllysine-residue succinyltransferase component of oxoglutarate dehydrogenase (DLST), malate dehydrogenase (MDH), succinyl-CoA synthetase alpha (SCSA), succinate-Coenzyme A ligase, beta subunit, putative (SCAL), succinate dehydrogenase [ubiquinone] iron-sulfur protein (SDIS), flavoprotein subunit of succinate dehydrogenase (FSSD), pyruvate carboxylase (PC), and fumarate hydratase (FH). Notably, ACN/IRP, a critical regulatory enzyme in the TCA cycle, had the highest number of Kla sites (*n* = 8). Five Kla sites (K176, K276, K420, K455, and K514) on the pyruvate dehydrogenase complex, which are distributed in the apicoplast to drive *de novo* fatty acid biosynthesis in the parasite [27], were detected.

In addition, 14 proteins with 27 Kla sites were associated with oxidative phosphorylation (PATHWAY: map00190) (Table S11), including ubiquinol cytochrome c oxidoreductase, ATP synthase beta subunit ATP-B, type I inorganic pyrophosphatase, ATP synthase F1, delta subunit protein, vacuolar ATP synthase subunit, ATPase synthase subunit alpha (putative), ATP synthase F1 gamma subunit, and copper chaperone COX17-1 (putative).

### Invasion-associated proteins of ***T***. ***gondii*** are lactylated

Like most protozoan parasites, *T*. *gondii* utilizes a unique movement machinery involving the actin–myosin complex for gliding to invade host cells [28]. We systemically analyzed the lactylated proteins involved in parasite gliding motility, activation of host cell entry, and egress from the infected cells (Table S12). Six myosins (myosin A, myosin C, myosin D, myosin F, myosin light chain 2, and myosin head domain-containing protein) containing 19 Kla sites and two actins (including actin-depolymerizing factor ADF and actin ACT1) with 7 Kla sites were detected. *T*. *gondii* typically contains three secreted organelles: rhoptries (RONs and ROPs), micronemes (MICs), and dense granules (GRAs), which are indispensable for the invasion and survival in host cells [29], [30]. We detected various degrees of lactylation in MIC1 (K157), MIC2 (K630), RON6 (K1170), ROP9 (K79, K138, K144, and K347), ROP18 (K202), GRA12 (K74), and rhoptry proteins (K954 and K956). In addition, nine Kla sites were detected on three calcium-dependent protein kinase family proteins (CDPKs), including CDPK1 (K59, K80, K93, K341, and K350), CDPK2A (K165, K729, and K777), and CDPK9 (K5), all of which facilitate parasite gliding motility, cell invasion, egress, intracellular development, and reproduction [31].

### Lactylated proteins are involved in carbon metabolism, spliceosome formation, aminoacyl-tRNA biosynthesis, and ribosome biogenesis

A total of 300 lactylated proteins with 2675 nodes were mapped to the database using STRING database (confidence score > 0.7), and the Cytoscape software was used to obtain a comprehensive view of the PPI network of the lactylated proteins. As observed in the network, lactylated proteins were significantly associated with various biological pathways, such as ribosome, carbon metabolism, spliceosome, aminoacyl-tRNA biosynthesis, and ribosome biogenesis (Figure S8; Table S13). The significant hub proteins included bifunctional dihydrofolate reductase-thymidylate synthase (TGME49_249180), heat shock protein HSP70 (TGME49_273760), elongation factor 1-gamma (TGME49_300140), proliferation-associated protein 2G4 (TGME49_279390), pre-mRNA-splicing factor CEF1 (TGME49_275480), and fibrillarin (TGME49_311430), which formed a dense PPI network.

### Histone lactylation is profoundly identified in ***T***. ***gondii***

**Figure 3:**
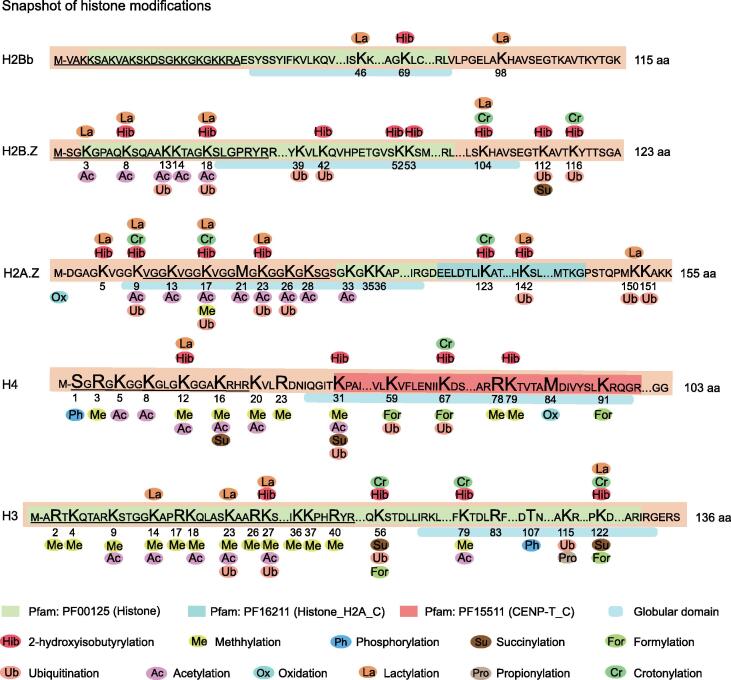
**Multiple PTMs occur at the same histone sites** The numbers indicate the specific locations of the modification sites on histones. Ellipses with different colors represent different types of PTMs. The different colored boxes represent the different domains. The horizontal light blue line describes the characteristics of amino acids that mediate PPI or biological processes (specific information from the Universal Protein Resource database, https://www.uniprot.org/uniport). PTM, post-translational modification; PPI, protein–protein interaction.

**Figure 4:**
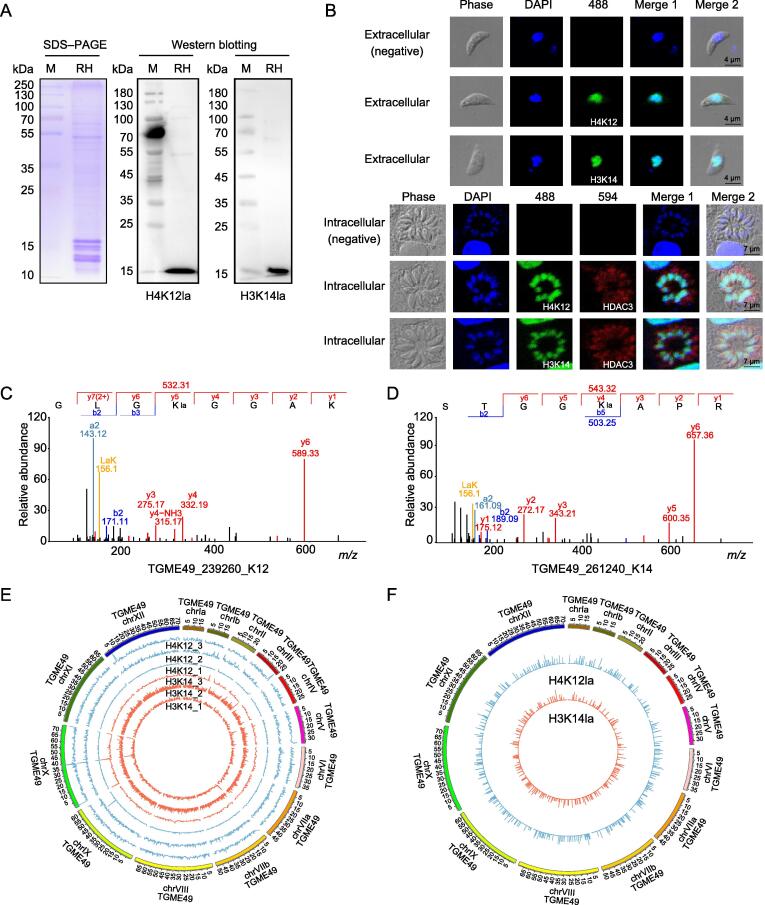
**H4K12 and H3K14 of*T. gondii*are identified as histone lactylation sites** **A.** Western blotting of histones H4K12la and H3K14la. **B.** Indirect IFA of histones H4K12la and H3K14la in the intracellular and extracellular stages of the parasite. Rabbit IgG was used as the negative control. **C.** and **D.** MS/MS spectra of histone H4K12la (C) and H3K14la (D) obtained from *T*. *gondii*. The a and b ions refer to the N-terminal part of the peptide, and the y ion refers to the C-terminal part of the peptide. **E.** Distribution of the binding regions of histone lactylation sites (H4K12la and H3K14la) on the chromosome shown in circos map. The outermost circle shows the chromosomes and the inner circles show the distribution trend for each sample. Data represent three independent experiments. **F.** Distribution of overlapping H4K12la and H3K14la peaks in the whole genome. The length of the circled bars represents the degree of enrichment and the position within the circle corresponds to the position of the peaks. ChIP-seq, chromatin immunoprecipitation sequencing.

Histone modification is one of the most important mechanisms in epigenetic regulation of eukaryotic genes [32]. Here, a comprehensive map of histone PTMs in the *T*. *gondii* RH strain was constructed (Figure 3, Figure S9). The analysis identified 17 Kla sites (Table S14) in five histones: H2A.Z (K5, K9, K17, K23, K142, and K150), H2B.Z (K3, K8, K18, and K104), H2Bb (K46 and K98), H3 (K14, K23, K27, and K122), and H4 (K12). In addition, 163 (86.7%) of the PTM sites were concentrated in H2B.Z, H2A.Z, H2Ba, H3, and H4, and most of the PTM sites were located at the N-termini of H2A.Z, H4, H2B.Z, and H3, but not H2Ba (at its C-terminus). Notably, more than four PTM types were detected in H2BaK70, H2BaK99, H2BaK111, H2B.ZK18, H2A.ZK9, H2A.ZK17, H2A.ZK23, H3K23, H3K27, H3K56, H3K79, H3K122, H4K12, H4K31, and H4K67. Although histone modifications are essential in the regulation of gene transcription and expression [33], [34], information regarding H4K12la and H3K14la remains limited. To further confirm the presence of H4K12la and H3K14la, we performed Western blotting and IFA analysis using anti-lactyl-histone H4 (Lys12) and anti-lactyl-histone H3 (Lys14) antibodies. Western blotting showed that the H4K12la and H3K14la signals co-migrated with bands of approximately 11 kDa and 15 kDa, respectively, which were consistent with the sizes of histones H4 and H3 (Figure 4A). We also confirmed, using IFA, that H4K12la and H3K14la were mainly distributed in the nuclei of the intracellular and extracellular stages (Figure 4B). The MS/MS spectra of the Kla sites of H3K14 and H4K12 are shown in Figure 4C and D, respectively (detailed spectral data are listed in Table S14).

Furthermore, we investigated the binding regions of H3K14la and H4K12la on chromosomes using chromatin immunoprecipitation sequencing (ChIP-seq), and data quality control met these requirements (Table S15). A total of 1779, 4011, and 1153 peaks were identified in the genomic binding regions of H3K14la in the three ChIP-seq experiments. Additionally, 2219, 2292, and 2134 peaks were identified in the H4K12la ChIP-seq assays (Table S16). Three biological replicates of ChIP-seq for H4K12la and H3K14la were performed, and the distribution of all peaks mapping the genome were shown using a circos plot (Figure 4E). Analysis of the average enrichment signals of H4K12la and H3K14la in all gene regions showed that they were mainly distributed around the transcriptional start site (TSS) regions (Figure S10A and B). The unique peaks in the binding regions of H4K12la and H3K14la were distributed on all 14 chromosomes of *T*. *gondii*, with TGME49_chrX, TGME49_chrVIII, and TGME49_chrXII being the most widely distributed (Figure 4F, Figure S10C and D).

The genomic distribution of H3K14la- and H4K12la-enriched regions can be divided into six categories: 5′-UTR, 3′-UTR, exon, intergenic, intron, and promoter. The peaks of H3K14la covered a large proportion of exons (40.64%) and promoters (37.91%), whereas only 12.37% were found in the intron regions (Figure S11A). Similar to the distribution of the peaks of H3K14la, H4K12la was also found mainly in exons (50.68%) and promoters (34.90%) (Figure S11B). We performed an overlap analysis on the genes regulated by H3K14la and H4K12la from the three biological replicates, and found that 195 genes were associated with H3K14la and 329 genes with H4K12la (Figure S11C and D). In addition, KEGG enrichment analysis showed that the genes regulated by H3K14la were enriched in the necroptosis pathway (Figure S11E; Table S17). Genes associated with H4K12la were mainly enriched in the cell cycle, ribosome biogenesis, phosphatidylinositol signaling system, and autophagy pathways (Figure S11F; Table S18). The overlap analysis also revealed that 147 and 281 specific genes were modulated by H3K14la and H4K12la, respectively (Figure S12A). The TSS enrichment analysis with the sequences associated with H3K14la and H4K12la showed that the sequences were also enriched around the TSS (3 kb upstream of TSS) and transcription end site (TES) regions (Figure S12B and C). These results suggest that histones H4K12la and H3K14la may actively regulate gene transcription in *T*. *gondii*.

Notably, many genes associated with H3K14la code for proteins in the kinesin complex, which are related to microtubule motor activity, thus participating in the microtubule movement pathway (Figure S13A; Table S19). The proteins encoded by genes associated with H4K12la were predominantly involved in ATP and protein binding, which are closely associated with neurotransmitter transport pathways (Figure S13B; Table S20). Furthermore, the genes regulated by both H3K14la and H4K12la were associated with the activity of a variety of enzymes, including ubiquitin-protein transferase, protein serine/threonine kinase, phosphotransferase, and acid-amino acid ligase, thus participating in the cellular protein modification process and microtubule-based motor pathways (Figure S13C; Table S21).

### Phosphofructokinase II is an important regulator of lactylation

**Figure 5:**
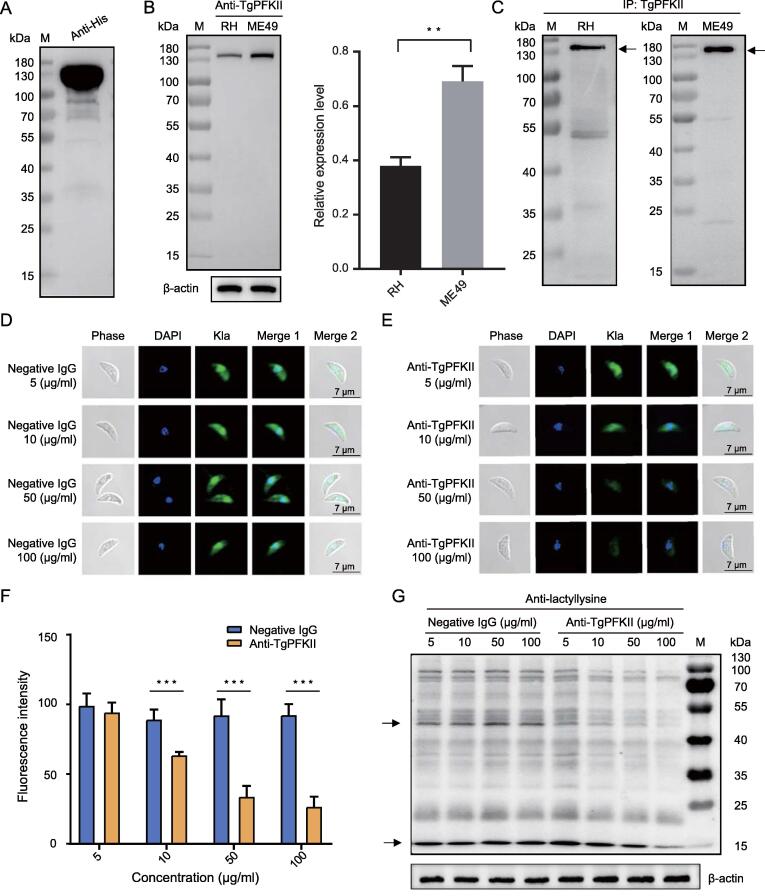
**Anti-TgPFKII antibodies reduced protein lactylation** **A.** The purified His-tagged TgPFKII was verified by Western blotting using the anti-His antibody. **B.** Western blotting analysis of TgPFKII expression levels (131 kDa) in *T*. *gondii* RH and ME49 using the anti-TgPFKII antibody. β-actin was used for normalization. The data are presented as mean ± standard deviation of three independent experiments (*, *P* < 0.05; **, *P* < 0.01; Student’s *t*-test). **C.** The soluble proteins derived from *T*. *gondii* RH and ME49 were immunoprecipitated with an anti-TgPFKII antibody, and the TgPFKII protein (arrow-headed) precipitated was detected by the anti-TgPFKII antibody. **D.** No effect on protein lactylation (green) with different concentrations of negative IgG (mouse IgG) analyzed by IFA. **E.** The anti-TgPFKII antibodies inhibited protein lactylation (green) in a concentration-dependent manner. **F.** Immunofluorescence intensity was statistically analyzed for the groups treated with the anti-TgPFKII antibody and the negative control antibody, respectively. Statistical significance analysis was performed using Student’s *t* test. All data are shown as mean ± standard deviation (*n* > 10). ***, *P* < 0.001. **G.** Western blotting assay was used to test the effect of different concentrations of anti-TgPFKII antibodies on the Kla level of *T*. *gondii* RH. β-actin was used for normalization. IP, immunoprecipitation; PFKII, phosphofructokinase II.

A recent study discovered that phosphofructokinase II (PFKII) is often induced in a hypoxic microenvironment, which greatly contributes to enhanced glucose metabolism, leading to increased levels of pyruvate [35]. To localize the expression of native PFKII in *T*.* gondii*, a hexa-histidine (His)-tagged TgPFKII fusion protein was generated and used to immunize animals to produce a TgPFKII-specific antibody (Figure 5A, Figure S14A and B). Then, the native TgPFKII was detected through Western blotting by the anti-TgPFKII antibody, and the expression of TgPFKII in the *T*. *gondii* ME49 strain were significantly higher than that in the *T*. *gondii* RH strain (Figure 5B and C). The IFA results showed that TgPFKII was widely distributed in multiple discrete compartments during the intracellular and extracellular stages (Figure S14C and D). In addition, seven proteins were identified to interact with TgPFKII using immunoprecipitation (IP) and MS analyses (Table S22), which include the proteasome 26S regulatory subunit (TGME49_313410), uncharacterized protein (TGME49_205320), uncharacterized protein (TGME49_240220), alanine-tRNA ligase (TGME49_219540), inner membrane complex protein IMC17 (TGME49_286580), elongation factor 2 family protein (TGME49_286080), and histidyl-tRNA synthetase (TGME49_280600). As shown in Figure S15, these proteins were differentially modified by lysine 2-hydroxyisobutyrylation (Khib), lysine crotonylation (Kcr), and Kla in the two *T*. *gondii* strains (Table S23), which is consistent with our previous findings [15].

The involvement of TgPFKII in protein lactylation was confirmed through the addition of TgPFKII-specific antibodies to parasite cultures, as previously described [36]. The antibodies were able to infiltrate free tachyzoites and were broadly disseminated in the cytoplasm, as illustrated by IFA and 3D laser scan technology (Figure S16A; Movies S1 and S2). The anti-TgPFKII antibody inhibited lactylation in a dose-dependent manner (Figure 5D–G), suggesting that TgPFKII is an important component of the *T*. *gondii* protein lactylation pathway. However, the anti-TgPFKII antibody had no effect on either *Plasmodium* (trophozoite) or *Trypanosoma* parasites, although it strongly reduced the lactylation in monkey kidney adherent epithelial (Vero) cells (Figure S16B–D).

### Antibodies to histone deacetylases and acetyltransferase impose significant effects on their enzymatic activities

**Figure 6:**
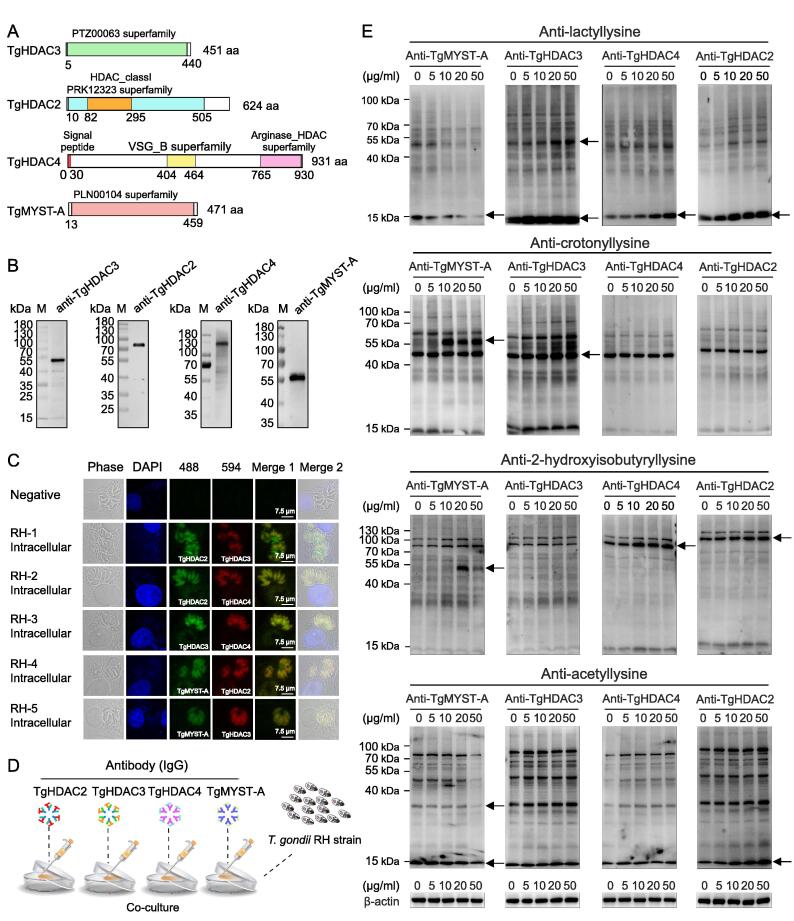
**Preliminary analysis of the function of TgHDAC2, TgHDAC3, TgHDAC4, and TgMYST-A in associated with protein lactylation** **A.** Sequence characteristics of TgHDAC2, TgHDAC3, TgHDAC4, and TgMYST-A. **B.** Western blotting analysis of the expression of native TgHDAC2 (71 kDa), TgHDAC3 (53 kDa), TgHDAC4 (101 kDa), and TgMYST-A (56 kDa) with protein-specific antibodies. **C.** Indirect IFA (co-localization) of TgHDAC2, TgHDAC3, TgHDAC4, and TgMYST-A. **D.** Flow chart on the effect of specific antibodies on PTMs. **E.** Modulation of anti-TgHDAC2, anti-TgHDAC3, anti-TgHDAC4, and anti-TgMYST-A antibodies on lactylation, crotonylation, 2-hydroxyisobutyrylation, and acetylation of *T*. *gondii*. Proteins with variation in modification level was pointed with an arrow. β-actin was used for normalization. HDAC2, HADC3, and HDAC4 are histone deacetylases; MYST-A is a kind of histone lysine acetyltransferase.

Histone deacetylases (HDACs) and histone lysine acetyltransferase (including MYST-A) control the acetylation state of histones in association with gene transcription and downstream biological events [37], [38]. In *T*. *gondii*, five genes coding for HDAC class I/II, including TgHDAC1–5, have been identified and potentially involved in histone PTMs [39]. These enzymes contain several functional domains: TgHDAC3 contains a PTZ00063 superfamily domain, TgHDAC2 contains a class I histone deacetylase structural domain (PRK12323 superfamily), TgHDAC4 contains arginase-like and histone-like hydrolases and a trypanosomal  variant surface glycoprotein (VSG) structural domain, and TgMYST-A contains a PLN00104 superfamily domain (Figure 6A). To localize the expression of the four proteins (TgHDAC2, TgHDAC3, TgHDAC4, and TgMYST-A) in *T*. *gondii*, we prepared specific antibodies, which were verified using Western blotting (Figure 6B, Figure S17A and B). TgHDAC3 and TgMYST-A were located in the cytosol and nuclei of parasites in both the extracellular and intracellular phases (Figure 6C, Figure S17C), as reported in previous studies [38], [40], [41]. Notably, TgHDAC3 was distributed more in the cytosol than in the tachyzoite nuclei. In addition, TgHDAC2 and TgHDAC4 were located in the cytosol and nuclei.

We then explored whether the four enzymes affected PTMs in tachyzoites *in vitro* (Figure 6D). The anti-TgHDAC2, anti-TgHDAC3, and anti-TgHDAC4 antibodies increased the overall level of protein lactylation, suggesting that these enzymes negatively regulate protein lactylation in *T*. *gondii*. And anti-TgHDAC3 antibody enhanced protein crotonylation, whereas anti-TgHDAC4 and anti-TgHDAC2 antibodies had no similar effect. In addition, anti-TgHDAC2 and anti-TgHDAC4 antibodies increased 2-hydroxyisobutyrylation of non-histone proteins. Furthermore, we also observed that the addition of anti-TgHDAC2 antibody in culture increased overall protein acetylation, and anti-TgHDAC3 only slightly increased non-histone protein acetylation. In contrast, anti-TgMYST-A antibodies reduced protein lactylation and acetylation. Notably, when the concentration of the anti-TgMYST-A antibody was greater than 20 μg/ml, the levels of 2-hydroxyisobutylation and crotonylation of non-histone proteins (approximately 55 kDa) increased significantly (Figure 6E).

We also examined whether TgHDAC2, TgHDAC3, TgHDAC4, and TgMYST-A affected the levels of H3K14la and H4K12la, as well as the expression of TATA-binding proteins, using Western blotting. The results revealed that anti-TgHDAC3 and anti-TgHDAC4 antibodies slightly increased the histone H3K14la levels (Figure S17D). In contrast, the level of H4K12la was increased by anti-TgHDAC2 and anti-TgHDAC3 antibodies, while the level of H4K12la remained unchanged in the presence of anti-TgHDAC4 and anti-TgMYST-A antibodies. The anti-TgMYST-A antibody significantly enhanced the expression of TATA-binding proteins (Figure S17D).

## Discussion

As an intracellular parasite, *T*. *gondii* utilizes multiple carbon sources to meet its own energy demands, and approximately 80% of internalized glucose in *T*. *gondii* is catabolized to lactate at the tachyzoite stage [25], [26], [42]. The intermediates and end products of its metabolic processes are recognized as essential signals associated with cellular activity and gene expression [43], [44]. Lactate was initially regarded as a metabolite of glycolysis and has recently been considered a signal that stimulates protein lactylation [20]. In this study, an atlas of the *T*. *gondii* RH strain lactylome was established for the first time, and this atlas contains 955 proteins with 1964 Kla sites. The flux of lactylation was close to that of crotonylation, which was second only to phosphorylation and 2-hydroxyisobutyrylation (Table S24). Our studies revealed that lactylated proteins were localized in diverse compartments (especially the mitochondria) and were markedly enriched in energy metabolism processes, such as oxidative phosphorylation, glyoxylate and dicarboxylate metabolism, glycolysis/gluconeogenesis, and the TCA cycle (Figure 2, Figure S4). These findings are in line with previous findings in *Trypanosoma brucei* and rice, where lactylation was identified as important for gene transcription and central carbon metabolism [45], [46]. Therefore, we focused on the association between lactylation and energy metabolism. We found that protein lactylation in glucose-rich culture medium was significantly higher than that in low-glucose medium (Figure S18A). Furthermore, inhibition of lactate generation using sodium dichloroacetate and oxamate resulted in less histone Kla, and inhibition of mitochondrial respiratory chain complex I using rotenone increased the level of histone Kla (Figure S18B–D), indicating that protein lactylation is positively correlated with glycolysis and mitochondrial metabolism in *T*. *gondii*. Moreover, we identified diverse lactylated rate-limiting enzymes involved in energy metabolism, including HK, PyK, and CSI. Interestingly, some glycolytic enzymes in *T*. *gondii* contain two isoforms that exhibit differential modification patterns at both tachyzoite and bradyzoite stages [3], [47]. Three Kla sites (K4, K109, and K136) on PyK1 and two Kla sites (K328 and K336) on PyK2 were identified. In addition, lactate dehydrogenase is an important enzyme for the conversion of pyruvate to lactate, and *T*. *gondii* expresses LDH1 only at the tachyzoite stage [48]. Here, we identified five Kla sites (K43, K94, K211, K218, and K322) in LDH1 of the *T*. *gondii* RH strain, which might be essential for balancing the levels of pyruvate and lactate. However, the biological significance of these different modifications requires further investigation.

Under eutrophic conditions, oxidative phosphorylation in mitochondria is the main pathway for ATP synthesis and is sufficient to support *T*. *gondii* reproduction. However, under physiological conditions, when ATP production is insufficient, LDH-mediated lactate fermentation is essential for energy supply [3]. Thus, lactylation may play a more central role in the regulation of energy metabolism in *T*. *gondii* under physiological conditions. Earlier studies reported that phosphorylation was mainly associated with signal transduction in *Toxoplasma* [49]; palmitoylation was more importantly linked to the process of *Toxoplasma* invasion of host cells [9]; ubiquitination was mainly associated with the regulation of the cell cycle transition of *T*. *gondii* [19]. In contrast to these PTMs, lactylation in *T*. *gondii* is highly correlated with energy metabolic processes.

In animal cells, glycolytic fluxes are highly regulated by fructose 2,6-diphosphate levels, and fructose-2-phosphate kinase mediates the formation of fructose-2,6-diphosphate from fructose-6-phosphate [50], [51]. The level of lactylation reduced dramatically after anti-TgPFKII antibody treatment in *T*. *gondii* and Vero cells, whereas it had no effect on either *Plasmodium* or *Trypanosoma* species (Figure 5, Figure S16), thus implying that the effect of enzyme-specific antibodies is parasite-dependent. In our previous study, TgPFKII was found to contain the highest number of modification sites in the glycolytic pathway [15]. Here, we further confirmed that TgPFKII was crotonylated and 2-hydroxyisobutyrylated (Figure S19), implying that these two forms of PTMs may modulate the activity of TgPFKII and protein lactylation.

There is growing evidence that enzymes such as HDACs and MYST-A are involved in the regulation of various cellular functions [41], [52]. In this study, we revealed that TgHDAC3 acts as a delactylase, decrotonylase, and deacetylase in tachyzoites, while TgHDAC2 regulates lactylation, acetylation, and 2-hydroxybutyrylation. We further demonstrated that TgHDAC4 has both delactylase and de-2-hydroxymethylase activities in tachyzoites. Notably, TgHDAC4 contains not only the arginase HDAC superfamily structural domain but also the VSG structural domain. The main function of the VSG structural domain is to assist the parasite in immune evasion [53], [54]. Unlike other HDACs, only TgHDAC4 contains a signal peptide (0–26 aa), therefore, it may be a secreted protein that not only functions as a modifying regulatory enzyme but also might be a molecule associated with host immune system interactions. Previous studies have shown that HDACs have effective delactylase activity [41], [55], which was confirmed in this study. Furthermore, TgMYST-A, a histone lysine acetyl transferase, was found to improve histone acetylation and lactylation levels, which might have a synergetic effect on the differentiation of *T*. *gondii*. Notably, the anti-TgMYST-A antibody increased the levels of crotonylation and 2-hydroxyisobutyrylation on non-histone proteins (55 kDa), which warrants further investigation.

**Figure 7:**
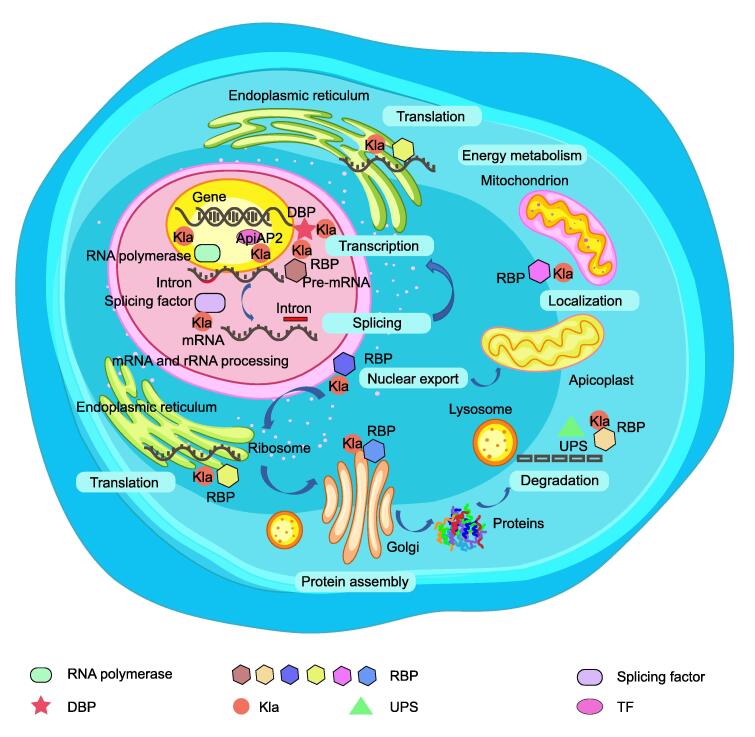
**A schematic diagram of the involvement of lactylation in gene regulation** The schematic diagram shows that lactylated proteins, such as TFs, RNA polymerases, DBPs, and RBPs, are involved in transcriptional and translational regulatory processes. The font inside the blue box indicates that lactylated RBPs may play a wide range of roles in various post-transcriptional processes at different cellular locations. Lactylated RBPs and splice factors are major players in splicing pre-mRNA into mature mRNA in the nucleus. In addition, lactylated RBPs can assist in the translation of mRNA into proteins in the ribosome, and some RBPs may be degraded by the UPS. TF, transcription factor; DBP, DNA-binding protein; UPS, ubiquitin–proteasome system; RBP, RNA-binding protein.

RBPs play indispensable roles in transcription, translation, and RNA processing in most eukaryotic cells [56], [57], [58]. Gene regulation and expression are mainly modulated by transcription-related proteins such as transcription factors (TFs), RBPs, splicing factors, RNA polymerases, and DNA-binding proteins (DBPs), which are crucial for the growth and reproduction of *T*. *gondii*. We summarized the lactylated proteins among the sets of TF-related proteins, RNA polymerase-related proteins, DBPs, and RBPs and found that they are associated with a range of processes, including gene transcription, RNA splicing, protein translation, and processing (Figure 7; Table S25). In total, 23 TFs, 7 RNA polymerases, 6 DBPs, and 27 RBPs with 49, 8, 8, and 62 Kla sites, respectively, were identified in *T*. *gondii*. Emerging evidence suggests that Apetala 2 (AP2) family proteins are the major TFs of *T*. *gondii*, and both activate and repress the bradyzoite developmental program [59]. Notably, the TF-related lactylated proteins mainly consist of the AP2 family, including AP2IX-6, AP2X-1, AP2XI-2, AP2XI-3, AP2VIII-7, AP2IV-5, AP2XII-5, AP2XII-1, AP2X-4, AP2X-7, AP2XI-4, AP2XI-5, AP2VIII-4, and AP2VIIa-9. Lactate production in host cells inhibits the differentiation of tachyzoites into bradyzoites [60]. As suggested by protein domain enrichment analysis (Figure S5), the RNA recognition motif is the top domain, and Kla is likely to coordinate RBP regulation, thus helping to precisely control gene expression at various life stages.

Histones are the primary structural components of chromatin, and histone PTMs play an indispensable role in epigenetic regulation of eukaryotic genes [32]. As shown in Figure 3, 17 Kla sites were detected in three core histones (H2Bb, H3, and H4) and two variant histones (H2A.Z and H2B.Z), which were mainly located in the N-terminal tail domains. Interestingly, most histone lysine sites undergo multiple PTMs rather than a single PTM. Notably, histones H2BbK46 and H2BbK98 were mono-modified in the globular domain, which might represent specific regulation of gene transcription by the PTMs. In human and mouse cells, 28 Kla sites have been identified on core histones that are closely associated with gene activation [22]. We found that the histone Kla sites of *T*. *gondii* are, in certain circumstances, similar to those of humans and mice. Among the 188 PTMs sites, 67 (35.6%) and 81 (43.1%) were distributed in the horizontal and globular domains, respectively, suggesting that PTMs co-regulate genes by altering the interactions between histones and DNA. Previous studies have reported that H4K12la is enriched in the promoters of genes associated with glycolysis and activates transcription in Aβ plaque-adjacent microglia, thereby increasing glycolytic activity [61]. The ChIP-seq data indicated that the binding regions of H4K12la and H3K14la on the chromosomes were concentrated near the TSS and were associated with cellular protein modification and microtubule motor processes in *T*. *gondii*. This is the first report to reveal an important relationship and function between H4K12la and H3K14la in *T*. *gondii* tachyzoites.

PTM crosstalk is important for the regulation of biological processes [62], [8], [63]. Here, we found that diverse PTMs occurred on the same lysine of a protein; for example, 392 lysines were commonly modified by Kla, Kcr, and Khib (Figure S20; Tables S26 and S27). And the Kcr-, Khib-, and Kla-modified proteins were mainly distributed in mitochondria, associated with ligase activity, and significantly enriched in processes such as ribosomal RNA monophosphate biosynthesis, tRNA metabolism, and protein translation (Figure S21; Table S28). Furthermore, three PTMs were related to the myosin head-motor domain, implying that these PTMs may functionally regulate proteins involved in host cell invasion by *T*. *gondii* (Figure S22A; Table S29). Additionally, Kcr-, Khib-, and Kla-modified proteins were enriched in the glycolysis/gluconeogenesis pathway, propanoate metabolism, the TCA cycle, aminoacyl-tRNA biosynthesis, RNA transport, oxidative phosphorylation, and ribosome processing (Figure S22B; Table S30). Therefore, multiple PTMs might regulate various biological processes.

In conclusion, we established the first comprehensive atlas of protein lactylation in the zoonotic parasite *T*. *gondii* and revealed that lactylation profoundly modulates proteins associated with energy metabolism and gene activation in various biological processes in the parasite (Figure S23). The data provide a new front for developing anti-parasite drugs.

## Materials and methods

### Experimental design and statistical rationale

The global Kla of the total proteins derived from the *T*. *gondii* RH strain was analyzed using a label-free method. Three biological replicates of protein lactylation were analyzed to evaluate the accuracy of the data obtained in the experiment (Table S1). In addition, qualitative analysis of lactylation was performed, and Fisher’s exact test was used to determine whether the data were statistically significant. Finally, R scripts were used for statistical tests and pattern generation.

### Experimental animals

Female Kunming mice (5–7 weeks old) and rats (200 g) were purchased from the Liaoning Changsheng Biological Technology Company (Changchun, China).

### Cultivation of *T*. *gondii*

*T*. *gondii* tachyzoites were cultured in a confluent monolayer of Vero cells and incubated at 37 °C with 5% O_2_ in high-sugar and low-sugar media (Catalog Nos. SC102-01 and SC104-01, Seven Biotech, Beijing, China) containing fetal bovine serum (Catalog No. 10099, Thermo Fisher Scientific, Waltham, MA) as previously described [64], [65].

### Parasite purification and protein extraction

Tachyzoites from the peritoneal fluid of *T*. *gondii* RH strain-infected mice were filtered through 5.0-μm nucleopore filters and collected through centrifugation in a centrifuge tube (Catalog No. CFT920150, Jet Bio-Filtration, Guangzhou, China). The Percoll (Catalog No. 17-0891-09, GE Healthcare, Uppsala, Sweden) gradient centrifugation method was used for separation and purification. Purified tachyzoites were washed using cold 1× phosphate-buffered saline (PBS) and sonicated three times in lysis buffer (8 M urea and 1% protease inhibitor), in the presence of a cocktail of protease inhibitors (Catalog No. HY-K0010, MCE, Monmouth Junction, NJ), using a sonicator (Catalog No. 01A566, Scientz, Ningbo, China). The lysates were centrifuged at 12,000 *g* (4 °C) for 12 min to remove insoluble cell fragments and the sample concentration was measured using a Bicinchoninic Acid (BCA) assay kit (Catalog No. P0010, Beyotime, Shanghai, China).

### Trypsin digestion of parasite proteins and high-performance liquid chromatography fractionation

The protein solutions were reduced at 56 °C for 30 min using 5 mM dithiothreitol, and alkylated at 37 °C for 15 min using 11 mM iodoacetamide in the dark. Subsequently, the protein samples were diluted using 100 mM tetraethylammonium bromide and trypsin was added for overnight digestion. The peptides were separated through high-pH reversed-phase separation using a Thermo Betasil C18 column (Catalog No. 70103-014001, Thermo Fisher Scientific).

### Antibody-based PTM enrichment and LC–MS/MS analysis

The peptides were dissolved in an IP buffer solution [100 mM NaCl, 1 mM ethylene diamine tetraacetic acid (EDTA), 50 mM Tris-HCl, 0.5% NP-40, pH 8.0], and the supernatant was transferred to pre-washed pan anti-lactyllysine antibody-conjugated beads (Catalog No. PTM-1401, PTM BIO, Hangzhou, China) and incubated overnight at 4 °C. The peptide bonds on the beads were eluted using 0.1% trifluoroacetic acid and vacuum dried. The resulting peptides were desalted using a C18 ZipTip (Catalog No. ZTC18S096, Millipore, Boston, MA) as previously described [22]. The remaining steps were performed as described previously [15].

### Database search

Secondary MS data were retrieved using the MaxQuant (v1.5.2.8) software from the ToxoDB 46.0 database (8322 sequences), and the false positive rate (FDR) caused by random matching through the anti-database was calculated. The remaining steps were performed as described previously [15].

### Preparation of specific antibodies

The genes coding for *T*. *gondii* HDAC2 (TGME49_249620), HADC3 (TGME49_227290), HDAC4 (TGME49_257790), MYST-A (TGME49_318330), and PFKII (TGME49_ 226960) were amplified from the genome of the *T*.* gondii* RH strain through polymerase chain reaction (PCR) using gene-specific primers (Table S31). The PCR products were cloned into pET-28a vector, respectively. The constructs containing the target genes were expressed in *Escherichia coli* BL21 (DE3) cells (Catalog No. CD601-02, TransGen Biotech, Beijing, China), and His-tagged recombinant proteins were purified according to the manufacturer’s instructions (Catalog No. CW0010S, CWBIO, Beijing, China) and evaluated using sodium dodecyl sulfate–polyacrylamide gel electrophoresis (SDS–PAGE) and Western blotting. Rats and mice (*n* = 10) were subcutaneously immunized four times using His-tagged recombinant proteins emulsified with Freund’s adjuvant. IgG was purified from the immune sera of immunized mice and rats using protein A (Catalog No. 17061801, Cytiva, Uppsala, Sweden) and protein G Sepharose 4 Fast Flow Resin (Catalog No. 17075601, Cytiva).

### Western blotting

*T*. *gondii* lysates (20 μg) were separated using SDS–PAGE, and the proteins were transferred to a polyvinylidene fluoride membrane (Catalog No. 162-0177, Bio-Rad, Hercules, CA). The membrane was blocked using 5% skim milk at 37 °C for 45 min and then incubated overnight at 4 °C with an anti-lactyllysine antibody (Catalog No. PTM-1401, PTM BIO). The membrane was washed four times with 1× PBS buffer and incubated with a secondary antibody at 37 °C for 50 min (Catalog No. 31430, Thermo Fisher Scientific). The procedure for the Western blotting assays of the recombinant proteins was the same as above (with an antibody dilution ratio of 1:200). Anti-TATA-binding protein and anti-β tubulin antibodies were purchased from PTM BIO (Catalog Nos. PTM-5007 and PTM-5028, PTM BIO).

### IFA

Tachyzoites were fixed on glass slides using pre-chilled paraformaldehyde for 10 min. After permeabilization using 0.1% Triton X-100 for 12 min, tachyzoites were blocked with 5% skimmed milk for 30 min and then incubated with an anti-lactyllysine primary antibody (Catalog No. PTM-1401, PTM BIO), which recognizes lactyllysine. The tachyzoites were subsequently incubated with Alexa Fluor-conjugated secondary antibodies (Catalog Nos. 31430 and 31460, Thermo Fisher Scientific) at 37 °C for 30 min, and the nuclei were stained as described previously [66], [67]. The negative control was the tachyzoites incubated with negative rabbit or mouse IgG (Catalog Nos. AG-0021 and AG-0051, Dingguo Biotechnology, Beijing, China). The parasites were stained with ProLong Gold Antifade Mountant (Catalog No. P36982, Invitrogen, Carlsbad, CA), and the results were observed using a confocal microscope (Catalog No. TCS-SP8, Leica, Wetzlar, Germany). IFAs for TgHDAC2, TgHDAC3, TgHDAC4, TgMYST-A, and TgPFKII were performed as described above.

To verify the permeability of antibodies to live *T*. *gondii*, anti-TgPFKII antibodies (1:200) were added to the medium, incubated with *T*. *gondii* for 3–4 h, and then fixed in cold methanol. The tachyzoites were permeabilized using 0.1% Triton X-100 for 20 min and washed twice with PBS. The Alexa Fluor 488-conjugated secondary antibodies were added directly and incubated at 37 °C for 40 min. The glass slide was washed thrice with 1× PBS buffer, and the nuclei were stained as previously described [66]. The intracellular signals were recorded as described above.

### Inhibition assay using anti-TgHDAC2, anti-TgHDAC3, anti-TgHDAC4, anti-TgMYST-A, and anti-TgPFKII antibodies

Specific antibodies, including anti-TgHDAC2, anti-TgHDAC3, anti-TgHDAC4, anti-TgMYST-A, and anti-TgPFKII antibodies, were tested for their effects on crotonylation, 2-hydroxyisobutyrylation, lactylation, and acetylation. Before complete release of *T*. *gondii* RH tachyzoites from Vero cells, antibodies of TgHDAC2, TgHDAC3, TgHDAC4, TgMYST-A, or TgPFKII were added at concentrations of 0, 5, 10, 20, and 50 µg/ml, respectively, to the cells and incubated at 37 °C for 4 h. The tachyzoites of *T*. *gondii* RH were centrifuged and rinsed with cold sterile 1× PBS buffer for 2 h. Total protein was extracted using a protein extraction kit (Catalog No. FNN0011, Thermo Fisher Scientific). The effect of the antibodies on PTMs was examined through Western blotting. The effect of a murine β-actin IgG (Catalog No. PTM-5018, PTM BIO) was used for normalization.

### IP experiment for TgPFKII

To identify the proteins interacting with TgPFKII, the tachyzoites of *T*. *gondii* were collected, purified, and sonicated three times for 5 s in lysis buffer (Catalog No. C500005, Sangon Biotech, Shanghai, China) containing protease inhibitors. Protein concentrations were determined using a BCA kit to ensure that the protein masses of the *T*. *gondii* RH and ME49 strains were equal. Anti-TgPFKII antibody (2 μg) was added to the cell lysates, and an equivalent amount of IgG from unimmunized animals was used as a negative control. The samples were mixed with overnight agitation at 4 °C. Then, 20 μl protein A or protein G beads were added and mixed for 1–3 h at 4 °C. The beads were precipitated through centrifugation at 4 °C for 30 s, followed by washing five times with 500 μl 1× lysis buffer. Finally, the sediment was heated at 100 °C for 10 min after being mixed with 20–40 μl 5× SDS sample buffer. The beads were precipitated through centrifugation, and the supernatants were subjected to Western blotting experiments with anti-lactyllysine, anti-2-hydroxybutyryllysine, and anti-crotonyllysine antibodies (Catalog Nos. PTM-1401, PTM-801, and PTM-502, PTM BIO). The immunoprecipitated proteins were dissolved in SDS–PAGE. Protein bands were excised and analyzed through MS using LC–MS/MS on Q Exactive HF (Thermo Fisher Scientific) as previously described [68].

### The effect of lactate metabolic inhibitors on histone lactylation

Before complete release of *T*. *gondii* RH tachyzoites from Vero cells, inhibitors were added at concentrations of 0, 5, and 20 mM, to the cells at 37 °C for 4 h. After 4 h, tachyzoites of *T*. *gondii* RH were centrifuged and rinsed with cold sterile 1× PBS buffer after 2 h. Total protein was extracted and protein lactylation of *T*. *gondii* RH was examined through Western blotting. Lactate inhibitors including sodium dichloroacetate (DCA), sodium oxalate, and rotenone were purchased from Selleck (Catalog Nos. S8615, S6871, and S2348, Selleck, Houston, TX).

### ChIP-seq

ChIP experiments were performed using anti-H4K12la and anti-H3K14la antibodies (Catalog Nos. PTM-1411 and PTM-1414, PTM BIO). *T*. *gondii* parasites were collected in centrifuge tubes and washed with 1× PBS buffer. After centrifugation and removing the supernatant, 1% formaldehyde in PBS buffer was added to cross-link DNA and proteins for approximately 10 min at 37 °C. Glycine was then added to stop the cross-linking reaction. PBS containing 0.5% bovine serum and protease inhibitor was added to wash parasites. The samples were collected through centrifugation, resuspended in 200 μl lysis buffer (1% SDS, 10 mM EDTA, 50 mM Tris-HCl, pH 7.5), and incubated on ice for 10 min. The lysates were sonicated, and one-tenth of the lysates were removed as an input control. The remaining lysates were immunoprecipitated by adding 4 μg of either anti-H4K12la or anti-H3K14la antibodies to ChIP dilution buffer (1% Triton, 150 mM NaCl, 2 mM EDTA, 20 mM Tris-HCl, pH 7.5), which was pre-incubated with protein A/G magnetic beads (Catalog No. 10003D, Invitrogen). The mixture was incubated overnight at 4 °C and then washed twice with Radio Immunoprecipitation Assay (RIPA) buffer (1 mM EDTA, 10 mM Tris, 0.1% SDS, 0.1% Triton X-100, 0.1% Na-deoxycholate), LiCl buffer (0.5% NP-40, 0.25 M LiCl, 0.5% Na-deoxycholate), and Tris-EDTA buffer.

The DNA fragments were eluted using an elution buffer (1% SDS, 0.1 M NaHCO_3_), purified through phenol–chloroform extraction, and precipitated using glycogen and ethanol. The ChIP-DNA fragments were used to construct libraries using the Illumina protocol, which were then sequenced using the Illumina sequencing platform (Illumina NovaSeq 6000, Illumina, San Diego, CA).

### ChIP-seq data filtration

Trimming of Illumina paired-end and single-end data (low-quality bases) was performed using the Trimmomatic software (v0.36). It works in conjunction with the FASTQ or gzipped FASTQ software. Through “simple” trimming, each adapter sequence is tested against the read, and if a sufficiently accurate match is detected, the read is trimmed appropriately.

### Sequence alignment

Clean reads were mapped to the genome of *T*. *gondii* using Bowtie2 (v2.3.4.3) with the default parameters. The alignment file (SAM) was transformed to a BAM file and filtered using the following criteria: (1) only retain unique aligned reads; (2) remove reads with low mapping qualities (< 30).

### Peak calling and peak annotation

The MACS2 software (v2.1.2) was used to identify TF-binding sites and to analyze genomic complexity to assess the significance of ChIP-enriched regions. The peaks were annotated using ChIPseeker (v2.16.0) and an R package for annotating ChIP-seq data. The software supports both comparative analysis of ChIP peak maps and annotations. Peaks from three experiments annotated with the same genes were combined into the final identified peak.

### GO annotation

Lactylated proteins were subjected to GO analysis. The process of GO annotation mainly converts protein IDs into UniProt IDs matched with GO IDs, and then retrieves the corresponding information from the UniProt-Gene Ontology Annotation (GOA) database.

### Domain annotation

Using InterProScan software and the corresponding InterPro domain database, the identified proteins were annotated with protein domains.

### KEGG pathway annotation

The proteins were annotated using the KEGG Automatic Annotation Server (KAAS) tool and matched to the corresponding pathways using KEGG mapper.

### KOG annotation

The sequences of the lactylated proteins were matched using the KOG database (v2.2.28).

### Subcellular localization

The WoLF PSORT software was used to predict subcellular locations of the identified proteins.

### Motif analysis

The compositional characteristics of the ten amino acids upstream and downstream of the Kla site were analyzed using the MoMo software. The threshold for qualifying motifs was a peptide containing a particular sequence form greater than 20 and *P* < 0.000001. The difference score (DS) in the frequency of amino acids close to the modification site is shown in the heatmap.

### GO, KEGG, and protein domain enrichment analyses

For every group, a two-tailed Fisher’s exact test (*P* < 0.05) was used to assess the statistical significance of the results from the enrichment analyses.

### PPI network analysis

All lactylated proteins were matched against the STRING database (v11.0) to obtain PPI relationships (high confidence > 0.7). The PPI network was visualized using the R package “networkD3”.

## Ethical statement

All animal manipulations in this study were performed in accordance with the Shenyang Agricultural University Animal Husbandry Guidelines, China. The Ethics Committee of Shenyang Agricultural University approved the animal experiments (Approval No. SYXK<Liao>2020-0001).

## Data availability

The MS proteomics data have been deposited into the ProteomeXchange Consortium via the PRIDE partner repository (ProteomeXchange: PXD022700), and are publicly accessible at http://proteomecentral.proteomexchange.org. The ChIP-seq data have been made available in the Sequence Read Archive (SRA: PRJNA791485), and are publicly accessible at https://submit.ncbi.nlm.nih.gov/subs/bioproject/. ChIP-seq data have been deposited in the Genome Sequence Archive [69] at the National Genomics Data Center (NGDC), Beijing Institute of Genomics (BIG), Chinese Academy of Sciences (CAS) / Chinese National Center for Bioinformation (CNCB) (GSA: CRA006903), and are publicly accessible at https://ngdc.cncb.ac.cn/gsa. The proteomics data reported in this study have been deposited into the Open Archive for Miscellaneous Data [69] at the NGDC, BIG, CAS / CNCB (OMIX: OMIX001139), and are publicly accessible https://ngdc.cncb.ac.cn/omix.

## Competing interests

The authors have declared no competing interests.

## CRediT authorship contribution statement

**Deqi Yin:** Writing – original draft, Investigation, Validation, Data curation. **Ning Jiang:** Validation, Supervision. **Chang Cheng:** Validation. **Xiaoyu Sang:** Software. **Ying Feng:** Formal analysis. **Ran Chen:** Software. **Qijun Chen:** Conceptualization, Methodology, Data curation, Writing – review & editing, Supervision. All authors have read and approved the final manuscript.

## Supplementary material

Supplementary video 55 **The permeability of anti-TgPFKII IgG to live *T. gondii* was verified using 3D laser scan technology (Sample 1)**

Supplementary video 56 **The permeability of anti-TgPFKII IgG to live *T. gondii* was verified using 3D laser scan technology (Sample 2, irrelative antibody control)**

Supplementary Figure S1 **Basic information of LC-MS/MS data of lysine lactylation A.** Flow chart of proteomics analysis. **B.** Mass error distribution of all lactylated peptides. **C.** Distribution of lysine lactylated peptides. **D.** The distribution of lysine lactylation sites in each protein. HPLC, high performance liquid chromatography.

Supplementary Figure S2 **GO classification analysis of lactylated proteins** Distribution of proteins corresponding to lactylation sites identified by GO functional annotations. GO, Gene Ontology.

Supplementary Figure S3 **GO-based enrichment analysis of the lactylated proteins** The GO-based enrichment analysis included cellular component (**A**), molecular function (**B**), and biological process (**C**) (Fisher’s exact test, *P* < 0.05).

Supplementary Figure S4 **DAG of each GO category generated with the lactylated proteins** The red circle indicates that the modified proteins were significantly (*P* < 0.01) enriched in the GO classification, the yellow circle indicates that the modified proteins were significantly (*P* < 0.05) enriched in the GO classification, and the blue circles indicate that the modified proteins were not significantly enriched in the GO classification. The lines with arrows represent the upper and lower hierarchical relationships in GO classification. DAG, directed acyclic graph.

Supplementary Figure S5 **RNA recognition motif associated with lysine lactylation** Domain enrichment analysis of lactylated proteins (Fisher’s exact test, *P* < 0.05). The color of the circle represents the *P* value. A redder color represents a more significant category.

Supplementary Figure S6 **Lactylated proteins involved in the spliceosome process** The orange circles represent the number of lactylation sites. The standard spliceosome is composed of five snRNPs, namely U1, U2, U4, U5, and U6 snRNPs, and several SAPs (KEGG pathway database: map03040). The detailed data is provided in Table S9. SAP, spliceosome-associated protein; snRNP, small nuclear ribonucleoprotein.

Supplementary Figure S7 **Lactylated enzymes of glycolysis/gluconeogenesis and the citrate cycle** The proteins highlighted in blue are modified by lactylation. The orange circles represent the number of lactylation sites. The detailed data are provided in Table S10. TCA, tricarboxylic acid.

Supplementary Figure S8 **Interaction network of the lactylated proteins** The size of the circle represents the different node degrees. Circles of different colors represent different pathways: ribosomes (red), ribosome biogenesis in eukaryotes (green), aminoacyl-tRNA biosynthesis (purple), spliceosomes (yellow), and carbon metabolism (light blue). Gray circles represent unclassified lactylated proteins.

Supplementary Figure S9 **Overview of PTM sites identified on histones** The number marked on the sequence indicates the location of the histone modification site. Ellipses with different colors represent different types of PTMs. The different colored boxes represent the different domains. A horizontal line describes the characteristics of a region (amino acid) that mediates PPIs or other biological processes (derived from the Universal Protein Resource database).

Supplementary Figure S10 **TSS and TES regions analysis of H3K14la and H4K12la A** and **B.** TSS and TES enrichment maps for H3K14la and H4K12la. All gene regions of *T. gondii* are mapped onto the horizontal axis. The graph shows the average enrichment degree of all reads. The vertical coordinates represent the average signal value (degree of enrichment). The horizontal coordinates represent gene regions. **C.** and **D.** Unique peak distribution of H3K14la and H4K12la in the chromosomes. TES, transcription end site; TSS, transcriptional start site.

Supplementary Figure S11 **KEGG enrichment analysis of genes associated with H3K14la and H4K12la A.** Statistical distribution of unique H3K14la peaks in genetic elements. **B.** Statistical distribution of unique H4K12la peaks in genetic elements. **C.** Overlapping peaks of H3K14la from three replicate experiments. **D.** Overlapping H4K12la peaks from three replicate experiments. **E.** KEGG enrichment analysis of genes associated with H3K14la. **F.** KEGG enrichment analysis of genes associated with H4K12la. UTR, untranslated regions; rawP, enrichment significance (*P* value).

Supplementary Figure S12 **Overlapped analysis of genes associated with H3K14la and H4K12la A.** Venn diagram of the common H3K14la and H4K12la genes. **B.** Analysis of enrichment degree of unique genes associated with H3K14la. **C.** Analysis of enrichment degree of unique genes associated with H4K12la.

Supplementary Figure S13 **GO enrichment analysis of genes associated with H3K14la and H4K12la A.** GO enrichment analysis of unique genes related to H3K14la. **B.** GO enrichment analysis of unique genes related to H4K12la. **C.** GO Enrichment analysis of overlapped genes related to H3K14la and H4K12la.

Supplementary Figure S14 **Indirect IFA of TgPFKII A.** Purified His-tagged TgPFKII-His was analyzed by SDS–PAGE. **B.** The effect of IgG purification (TgPFKII) was identified using SDS–PAGE. **C.** Indirect immunofluorescence of TgPFKII in the tachyzoites (green). **D.** Indirect immunofluorescence of TgPFKII in the parasitophorous vacuole (green).

Supplementary Figure S15 **Analysis of the difference in Kla, Kcr, and Khib levels of TgPFKII binding proteins in the *T. gondii* RH and ME49 strains. A.** Western blotting analysis of lactylation in *T. gondii*. **B.** Analysis of the difference in the lactylation level of TgPFKII binding proteins. **C.** Analysis of the difference in the crotonylation level of TgPFKII binding proteins. **D.** Analysis of the difference in the 2-hydroxyisobutyrylation level of TgPFKII binding proteins. Kcr, lysine crotonylation; Khib, lysine 2-hydroxyisobutyrylation.

Supplementary Figure S16 **The effect of anti-TgPFKII antibody on lactylation of Trypanosoma, Plasmodium, and Vero cells A.** The permeability of anti-TgPFKII IgG to live *T. gondii* was verified by the indirect IFA. **B.** The study of the effect of anti-TgPFKII antibody on the lactylation of Trypanosome by indirect immunofluorescence. **C.** The study of the effect of anti-TgPFKII antibody on the lactylation of Vero cells by indirect immunofluorescence. **D.** The study of the effect of anti-TgPFKII antibody on the lactylation of Plasmodium by indirect immunofluorescence.

Supplementary Figure S17 **The effect of anti-TgHDAC2, anti-TgHDAC3, anti-TgHDAC4, and anti-TgMYST-A antibodies on H3K14la, H4K12la, and TATA protein A.** Western blotting analysis of recombinant proteins (TgHDAC2, TgHDAC3, TgHDAC4, and TgMYST-A). **B.** The effect of IgG purification was identified using SDS–PAGE. **C.** Indirect IFA of TgHDAC2, TgHDAC3, TgHDAC4, and TgMYST-A for the tachyzoite stage (green). Nuclei is colored in blue. **D.** The effect of different concentrations of antibodies (anti-TgHDAC2, TgHDAC3, TgHDAC4, and TgMYST-A) on H3K14la, H4K12la levels and the expression level of TATA protein was examined through western blotting.

Supplementary Figure S18 **The effect of inhibitors on lactylation A.** Lactylation level of *T. gondii* under high and low glucose conditions was measured through Western blotting. β-Actin was used as an internal reference standard. **B–D.** The effect of the sodium dichloroacetate (**B**), oxamate (**C**), and rotenone (**D**) on the lactylation level was detected through western blotting. β-Actin was used for normalization. DCA, sodium dichloroacetate.

Supplementary Figure S19 **TgPFKII is crotonylated and 2-hydroxyisobutyrylated A.** The lysine crotonylation of TgPFKII was detected through western blotting (131 kDa). **B.** The lysine 2-hydroxyisobutyrylation of TgPFKII was detected through western blotting (131 kDa).

Supplementary Figure S20 **Overlap analysis of Kla, Kcr, and Khib in the *T. gondii* RH strain A.** Overlap analysis of Kla, Kcr, and Khib sites. B. Overlap analysis of Kla, Kcr, and Khib proteins.

Supplementary Figure S21 **GO enrichment analysis of Kla, Kcr, and Khib in the *T. gondii* RH strain A.** GO functional enrichment analysis (cellular component) of Kla, Kcr, and Khib proteins. **B.** GO functional enrichment analysis (molecular function) of Kla, Kcr, and Khib proteins. **C.** GO functional enrichment analysis (biological process) of Kla, Kcr, and Khib proteins.

Supplementary Figure S22 **Domain and KEGG enrichment analysis of Kla, Kcr, and Khib proteins A.** Domain enrichment analysis of Kla, Kcr, and Khib proteins. **B.** KEGG pathway enrichment analysis of Kla, Kcr, and Khib proteins.

Supplementary Figure S23 **A schematic illustration of lactylation and metabolic regulation** The schematic highlights the relationship between glycolysis and protein lactylation. Figures were created using BioRender.com. ATP, adenosine triphosphate.

Supplementary Table S1 **Mass spectrometric identification of lysine lactylation**

Supplementary Table S2 **GO annotation information of lactylated proteins**

Supplementary Table S3 **Subcellular localization of lactylated proteins**

Supplementary Table S4 **GO enrichment analysis of lactylated proteins**

Supplementary Table S5 **Domain enrichment analysis of lactylated proteins**

Supplementary Table S6 **KEGG enrichment analysis of lactylated proteins**

Supplementary Table S7 **KOG classification of lactylated proteins**

Supplementary Table S8 **Motif analysis of lysine lactylation**

Supplementary Table S9 **Lactylated proteins related to spliceosome**

Supplementary Table S10 **Modified enzymes involved in energy metabolism**

Supplementary Table S11 **Lactylated proteins involved in oxidative phosphorylation**

Supplementary Table S12 **Lactylation of invasion-associated proteins**

Supplementary Table S13 **Lactylated proteins in the protein–protein interaction network**

Supplementary Table S14 **Basic information on histone lactylation sites**

Supplementary Table S15 **Quality control information for H3K14la and H4K12la**

Supplementary Table S16 **Identified peaks of H4K12la and H3K14la**

Supplementary Table S17 **KEGG enrichment analysis of genes associated with H3K14la**

Supplementary Table S18 **KEGG enrichment analysis of genes associated with H4K12la**

Supplementary Table S19 **GO enrichment analysis of genes associated with H3K14la**

Supplementary Table S20 **GO enrichment analysis of genes associated with H4K12la**

Supplementary Table S21 **GO enrichment analysis of common genes**

Supplementary Table S22 **Proteins interacting with TgPFKII**

Supplementary Table S23 **Mass spectral data for 2-hydroxyisobutylation and crotonylation of proteins interacting with TgPFKII**

Supplementary Table S24 **Proteomic studies of PTMs in *Toxoplasma gondii***

Supplementary Table S25 **Lactylated proteins involved in gene regulation, expression, and protein degradation**

Supplementary Table S26 **Analysis of overlapping sites of Kla, Kcr, and Khib**

Supplementary Table S27 **Analysis of overlapping proteins of Kla, Kcr, and Khib**

Supplementary Table S28 **GO enrichment analysis of Kla, Kcr, and Khib**

Supplementary Table S29 **Domain enrichment analysis of Kla, Kcr, and Khib**

Supplementary Table S30 **KEGG enrichment analysis of Kla, Kcr, and Khib**

Supplementary Table S31 **Primers used in this study**
