# Supplementary material for: Protein Lactylation and Metabolic Regulation of the Zoonotic Parasite Toxoplasma gondii
Source: Genomics Proteomics Bioinformatics. 2022 Oct 7;21(6):1163–81. doi: 10.1016/j.gpb.2022.09.010 (PMC11082259; doi:10.1016/j.gpb.2022.09.010)
Supplement: Supplementary Figure S1 — Basic information of LC-MS/MS data of lysine lactylation A. Flow chart of proteomics analysis. B. Mass error distribution of all lactylated peptides. C. Distribution of lysine lactylated peptides. D. The distribution of lysine lactylation sites in each protein. HPLC, high performance liquid chromatography. [file mmc1.pdf]

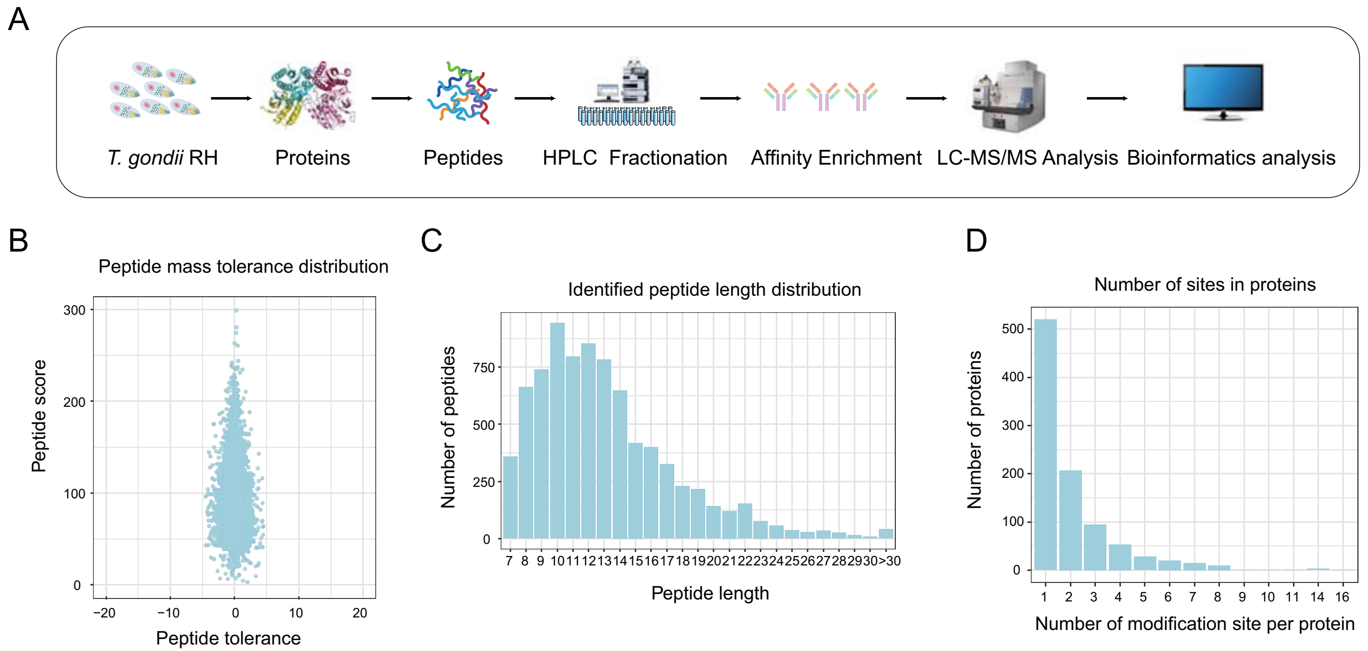
A
T. gondii RH
Proteins
Peptides
HPLC Fractionation
Affinity Enrichment
LC-MS/MS Analysis
Bioinformatics analysis
B
Peptide mass tolerance distribution
Peptide score
Peptide tolerance
C
Identified peptide length distribution
Number of peptides
Peptide length
D
Number of sites in proteins
Number of proteins
Number of modification site per protein
