# Supplementary figures and images for: Protein Lactylation and Metabolic Regulation of the Zoonotic Parasite Toxoplasma gondii
Source: Genomics Proteomics Bioinformatics. 2022 Oct 7;21(6):1163–81. doi: 10.1016/j.gpb.2022.09.010 (PMC11082259; doi:10.1016/j.gpb.2022.09.010)

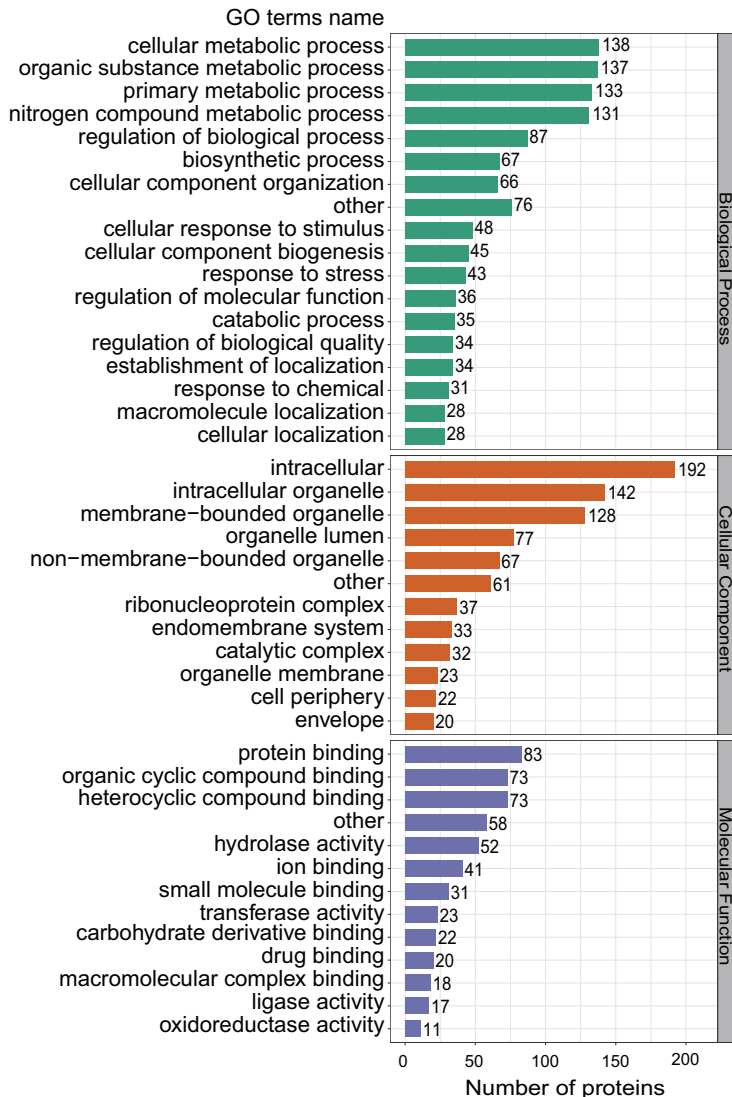

Supplement: Supplementary Figure S2 — GO classification analysis of lactylated proteins Distribution of proteins corresponding to lactylation sites identified by GO functional annotations. GO, Gene Ontology. [file mmc2.pdf]

A

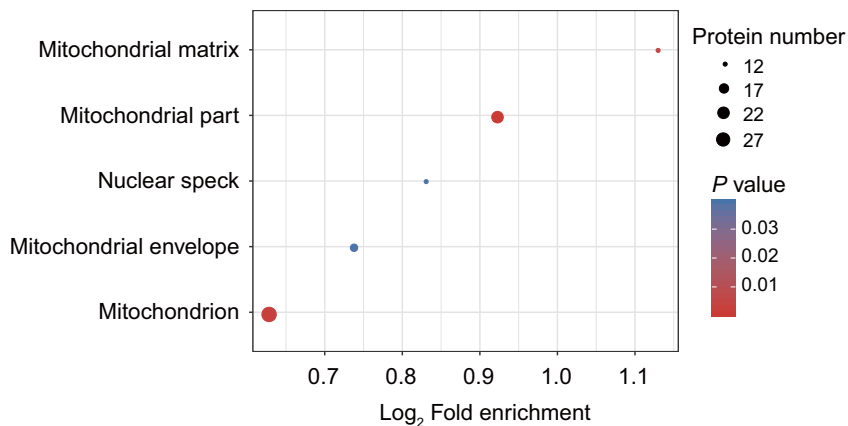

B

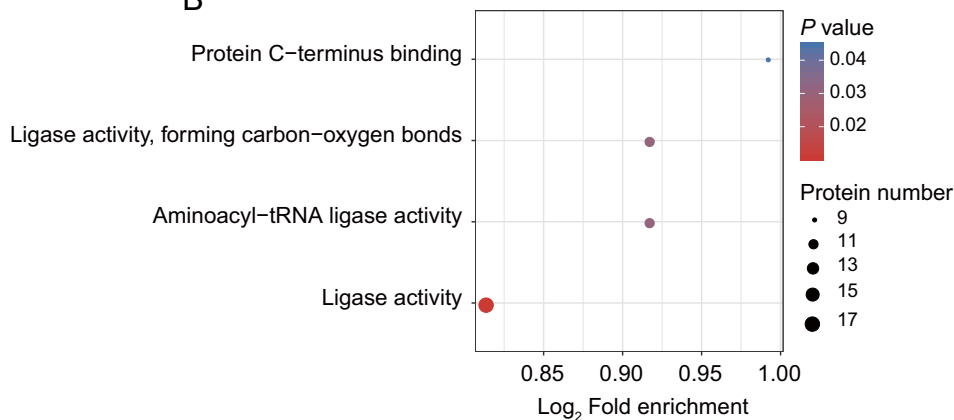

C

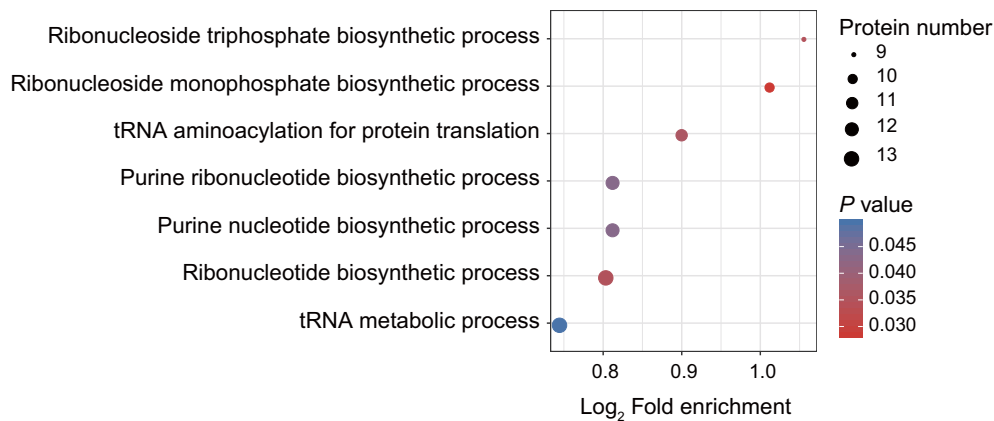

Supplement: Supplementary Figure S3 — GO-based enrichment analysis of the lactylated proteins The GO-based enrichment analysis included cellular component (A), molecular function (B), and biological process (C) (Fisher’s exact test, P < 0.05). [file mmc3.pdf]

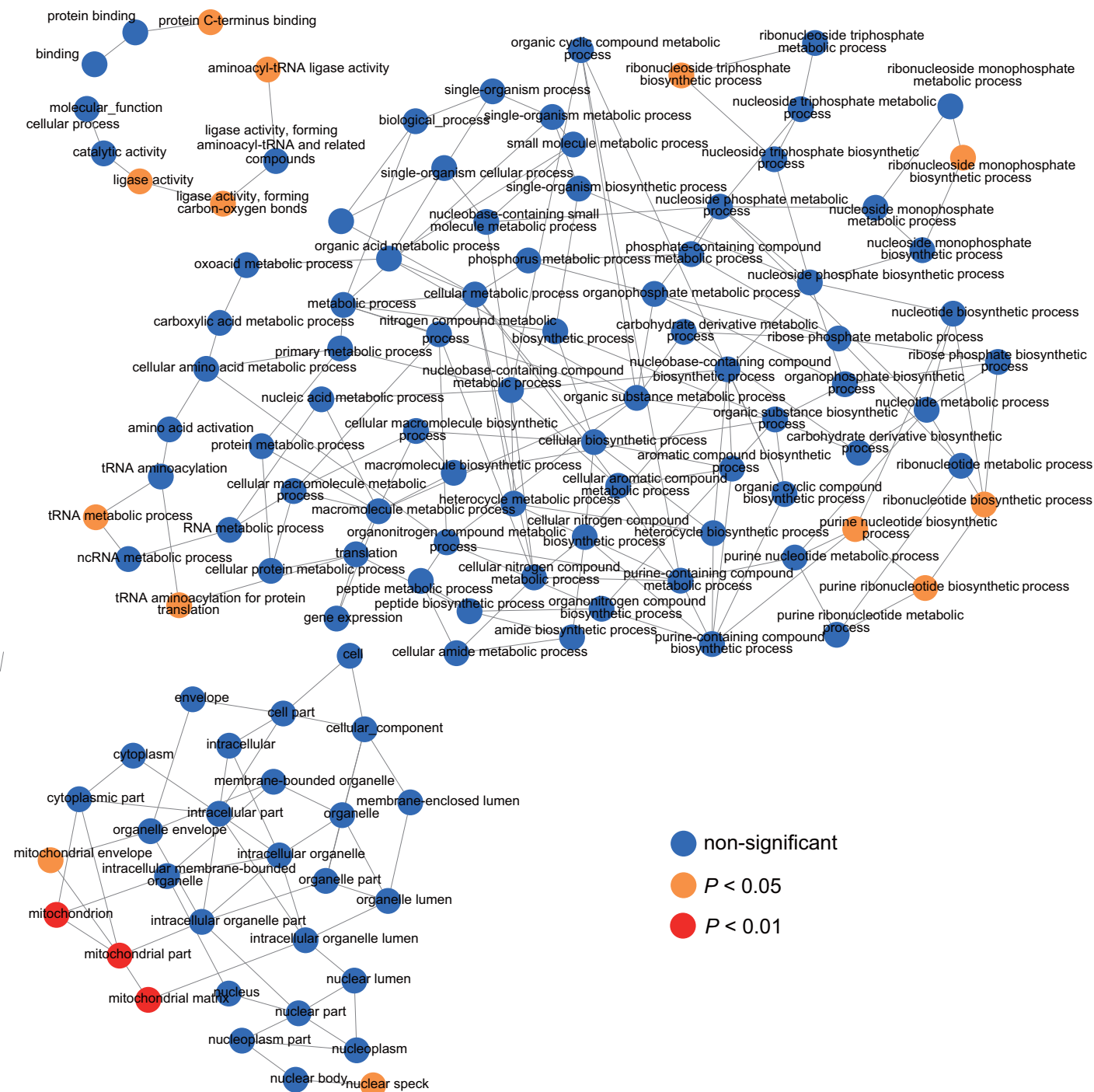

Supplement: Supplementary Figure S4 — DAG of each GO category generated with the lactylated proteins The red circle indicates that the modified proteins were significantly (P < 0.01) enriched in the GO classification, the yellow circle indicates that the modified proteins were significantly (P < 0.05) enriched in the GO classification, and the blue circles indicate that the modified proteins were not significantly enriched in the GO classification. The lines with arrows represent the upper and lower hierarchical relationships in GO classification. DAG, directed acyclic graph. [file mmc4.pdf]

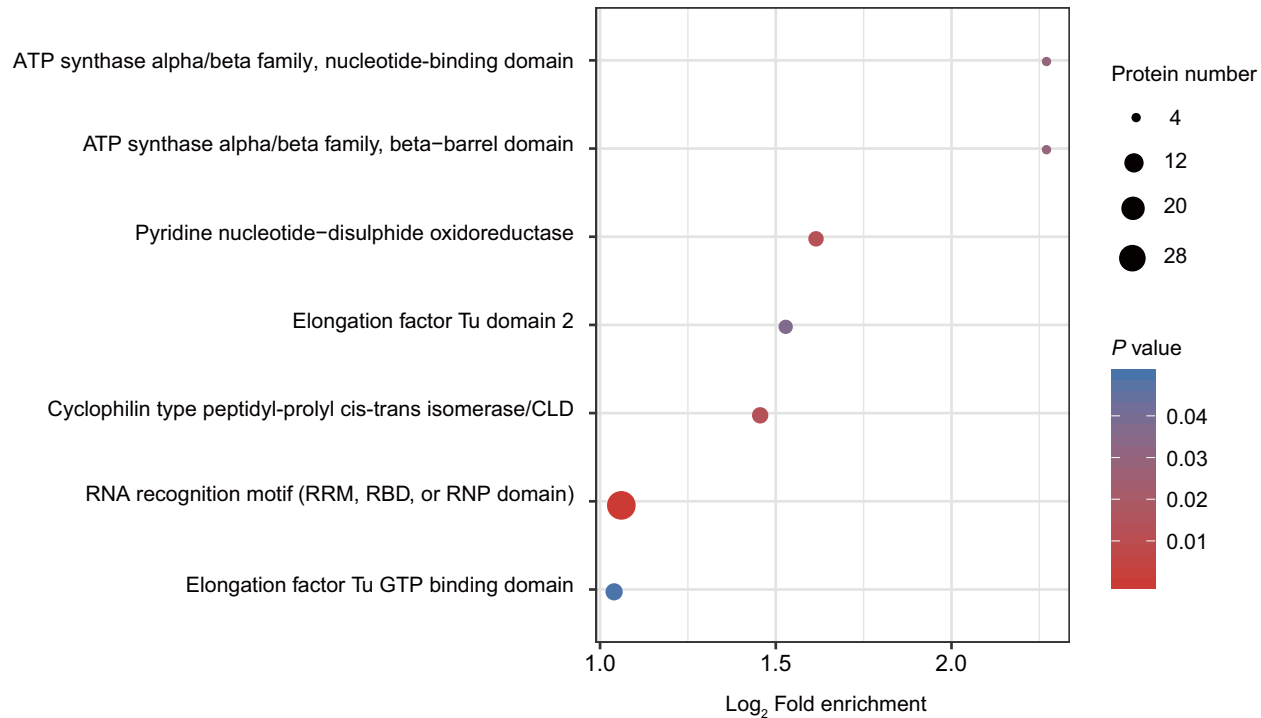

Supplement: Supplementary Figure S5 — RNA recognition motif associated with lysine lactylation Domain enrichment analysis of lactylated proteins (Fisher’s exact test, P < 0.05). The color of the circle represents the P value. A redder color represents a more significant category. [file mmc5.pdf]

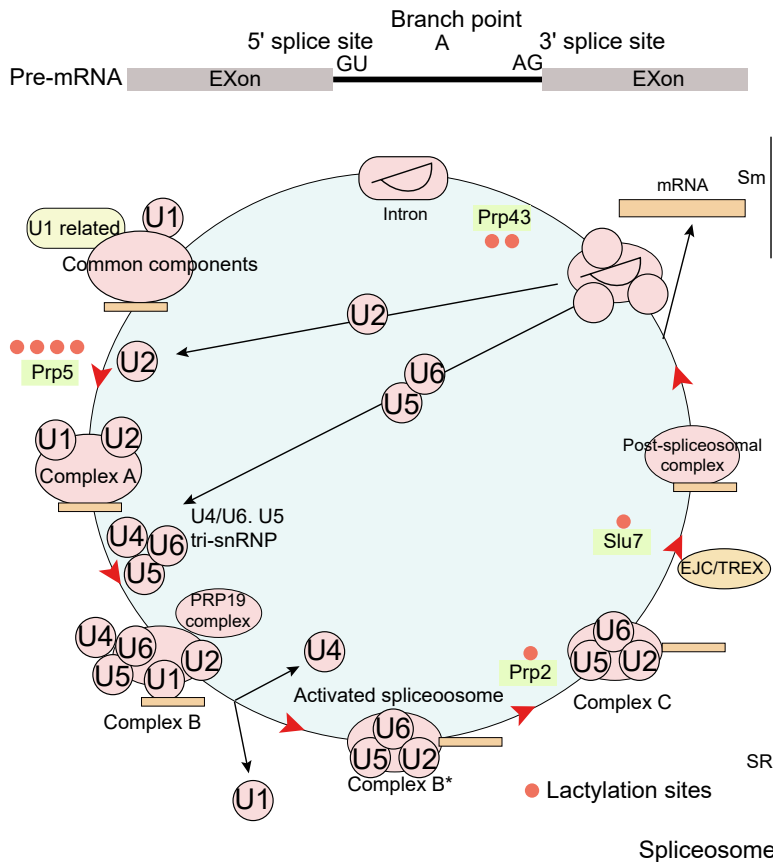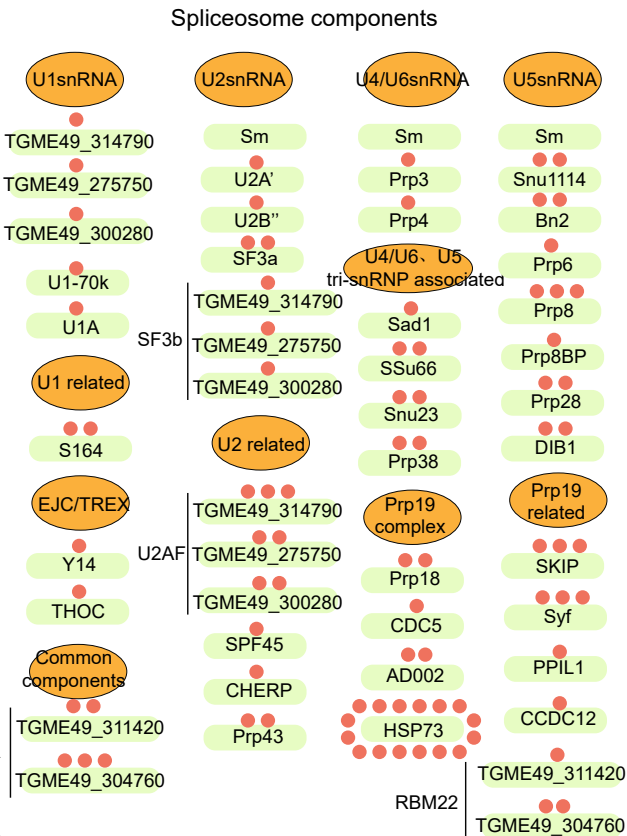

Supplement: Supplementary Figure S6 — Lactylated proteins involved in the spliceosome process The orange circles represent the number of lactylation sites. The standard spliceosome is composed of five snRNPs, namely U1, U2, U4, U5, and U6 snRNPs, and several SAPs (KEGG pathway database: map03040). The detailed data is provided in Table S9. SAP, spliceosome-associated protein; snRNP, small nuclear ribonucleoprotein. [file mmc6.pdf]

## Snapshot: Histone modifications

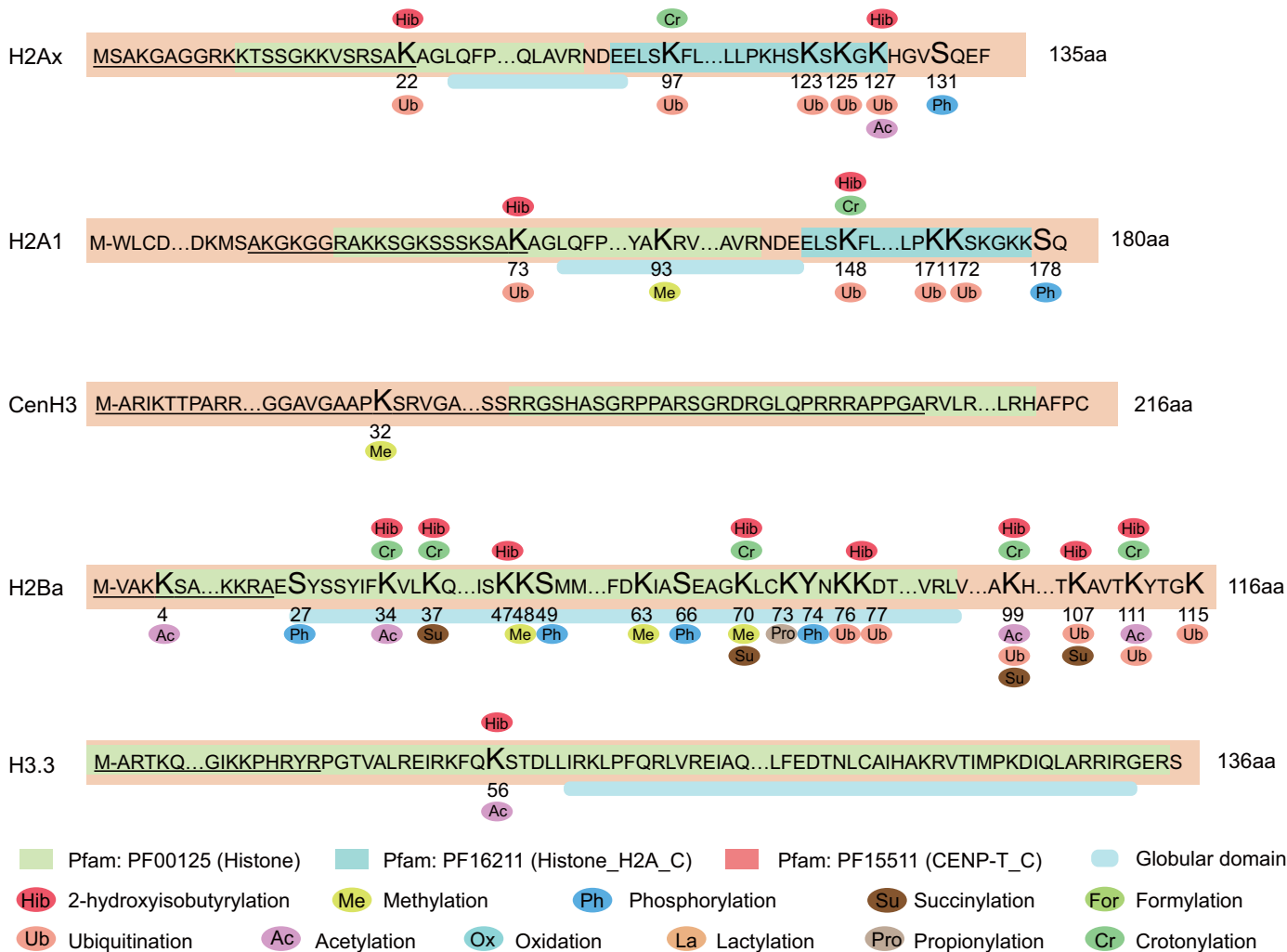

Supplement: Supplementary Figure S9 — Overview of PTM sites identified on histones The number marked on the sequence indicates the location of the histone modification site. Ellipses with different colors represent different types of PTMs. The different colored boxes represent the different domains. A horizontal line describes the characteristics of a region (amino acid) that mediates PPIs or other biological processes (derived from the Universal Protein Resource database). [file mmc9.pdf]

A

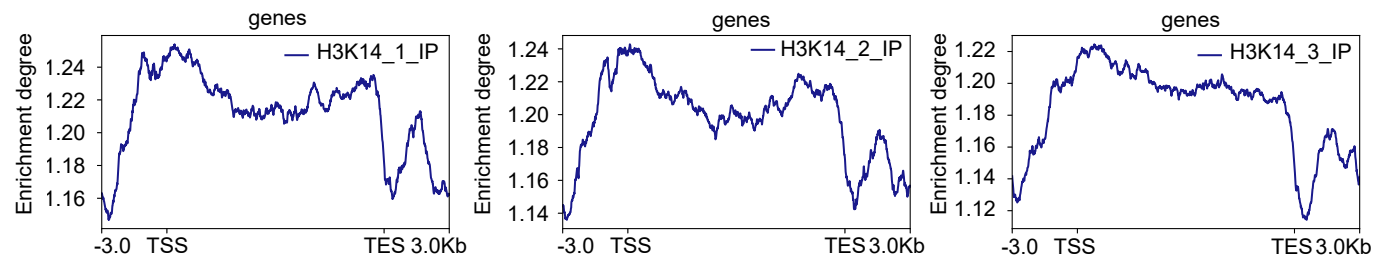

B

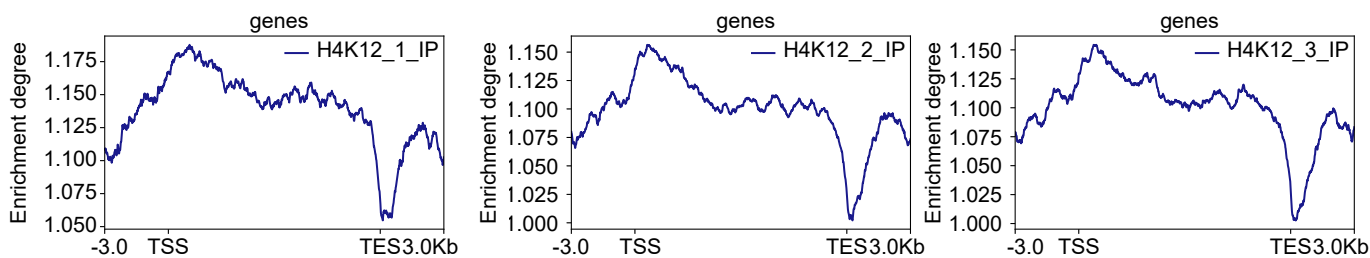

C

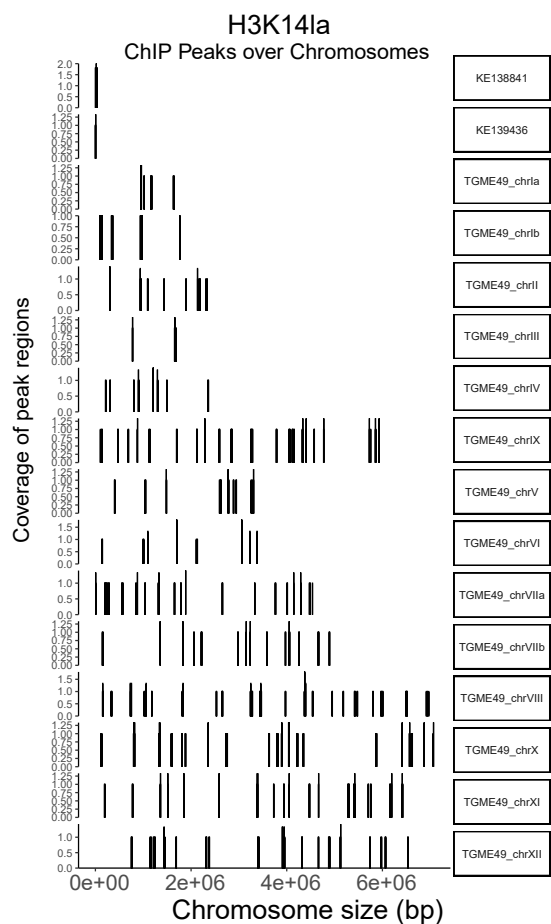

D

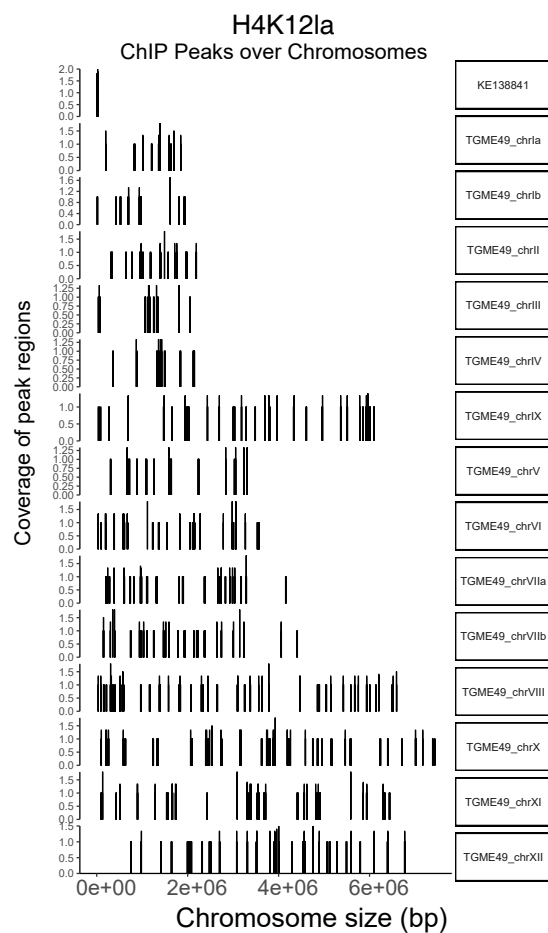

Supplement: Supplementary Figure S10 — TSS and TES regions analysis of H3K14la and H4K12la A and B. TSS and TES enrichment maps for H3K14la and H4K12la. All gene regions of T. gondii are mapped onto the horizontal axis. The graph shows the average enrichment degree of all reads. The vertical coordinates represent the average signal value (degree of enrichment). The horizontal coordinates represent gene regions. C. and D. Unique peak distribution of H3K14la and H4K12la in the chromosomes. TES, transcription end site; TSS, transcriptional start site. [file mmc10.pdf]

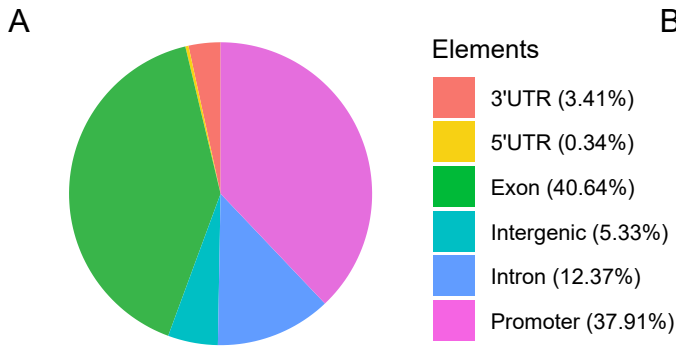

H3K14Ia

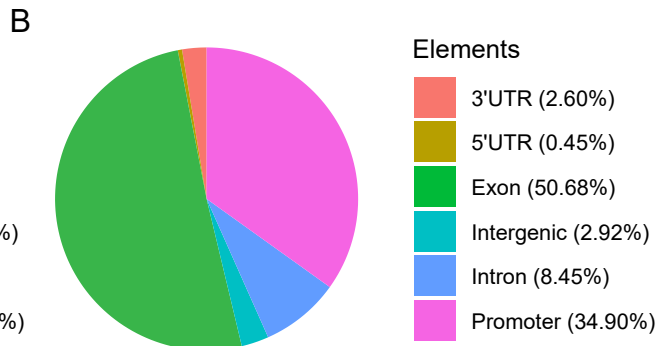

H4K12Ia

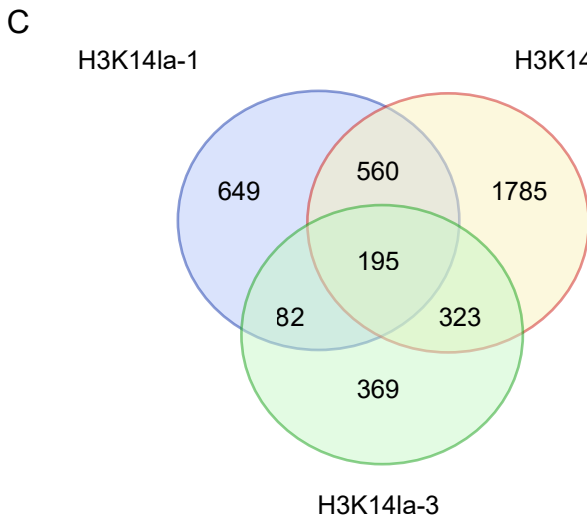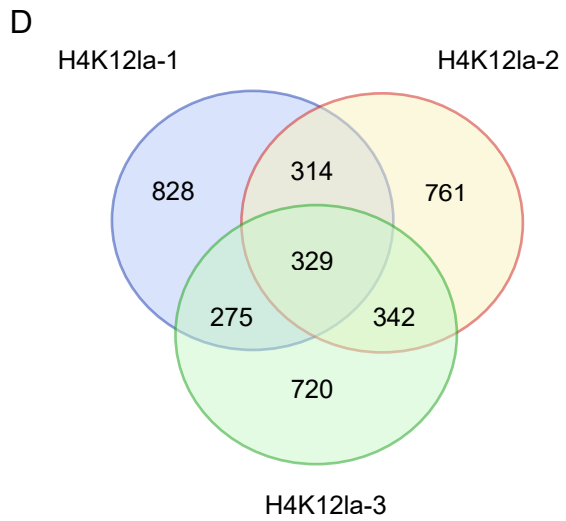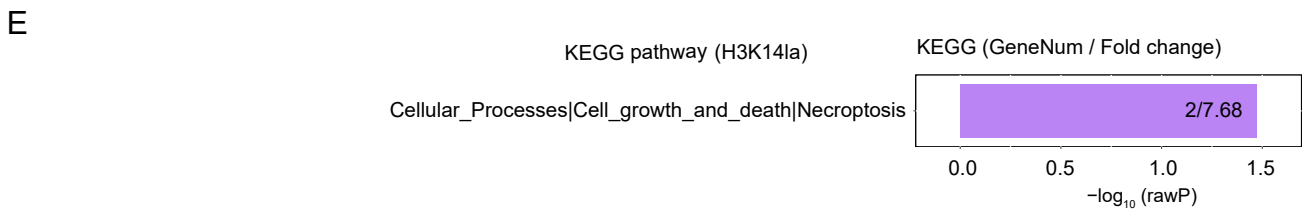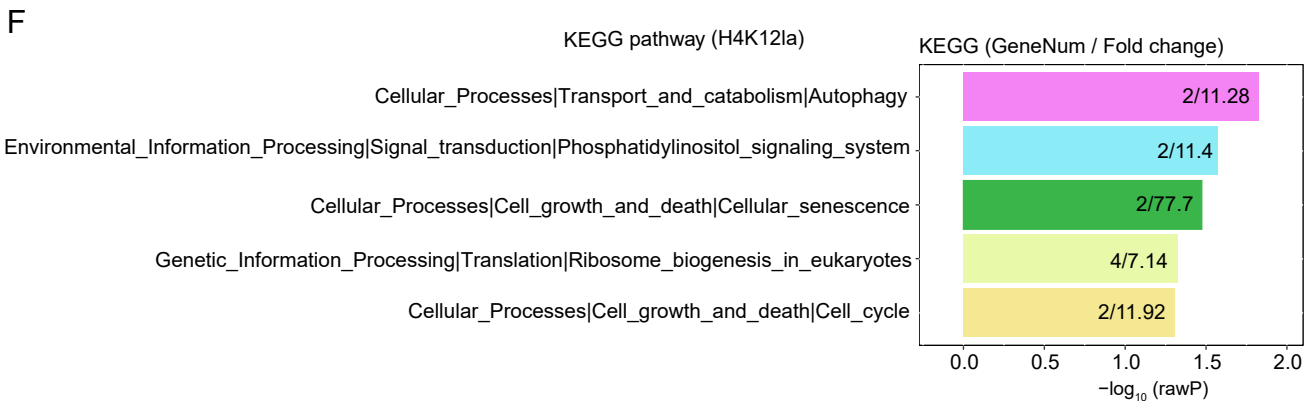

Supplement: Supplementary Figure S11 — KEGG enrichment analysis of genes associated with H3K14la and H4K12la A. Statistical distribution of unique H3K14la peaks in genetic elements. B. Statistical distribution of unique H4K12la peaks in genetic elements. C. Overlapping peaks of H3K14la from three replicate experiments. D. Overlapping H4K12la peaks from three replicate experiments. E. KEGG enrichment analysis of genes associated with H3K14la. F. KEGG enrichment analysis of genes associated with H4K12la. UTR, untranslated regions; rawP, enrichment significance (P value). [file mmc11.pdf]

**A**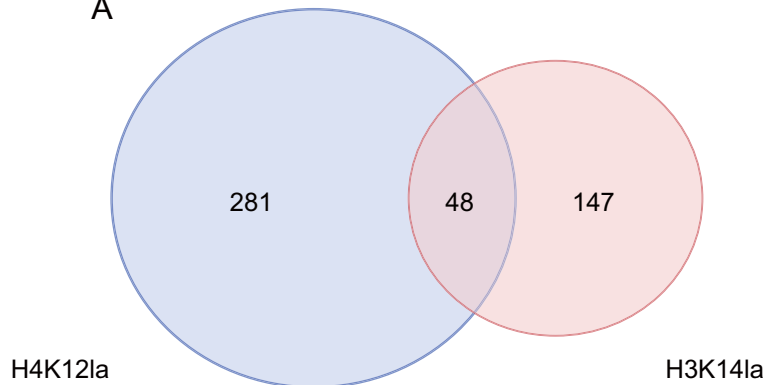**B****H3K14la**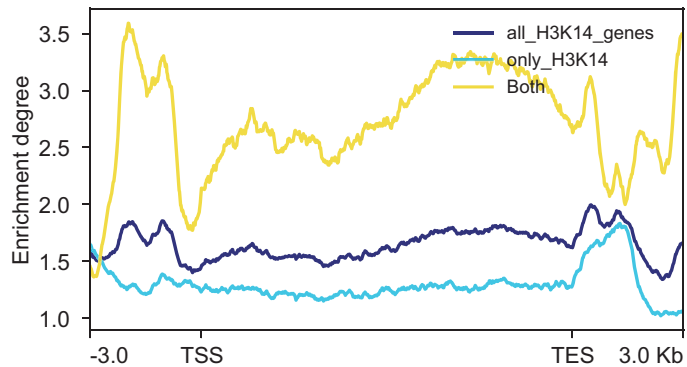**C****H4K12la**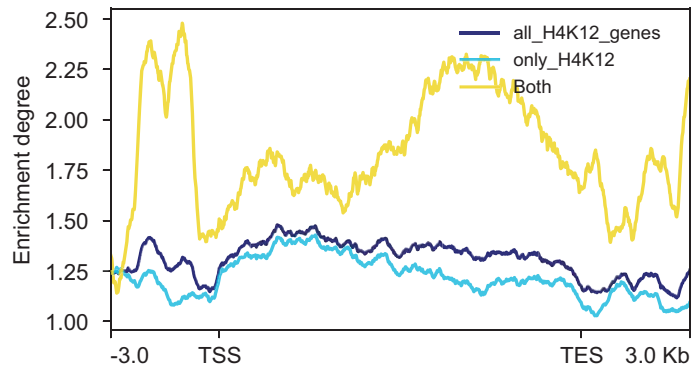

Supplement: Supplementary Figure S12 — Overlapped analysis of genes associated with H3K14la and H4K12la A. Venn diagram of the common H3K14la and H4K12la genes. B. Analysis of enrichment degree of unique genes associated with H3K14la. C. Analysis of enrichment degree of unique genes associated with H4K12la. [file mmc12.pdf]

A

GO enrichment

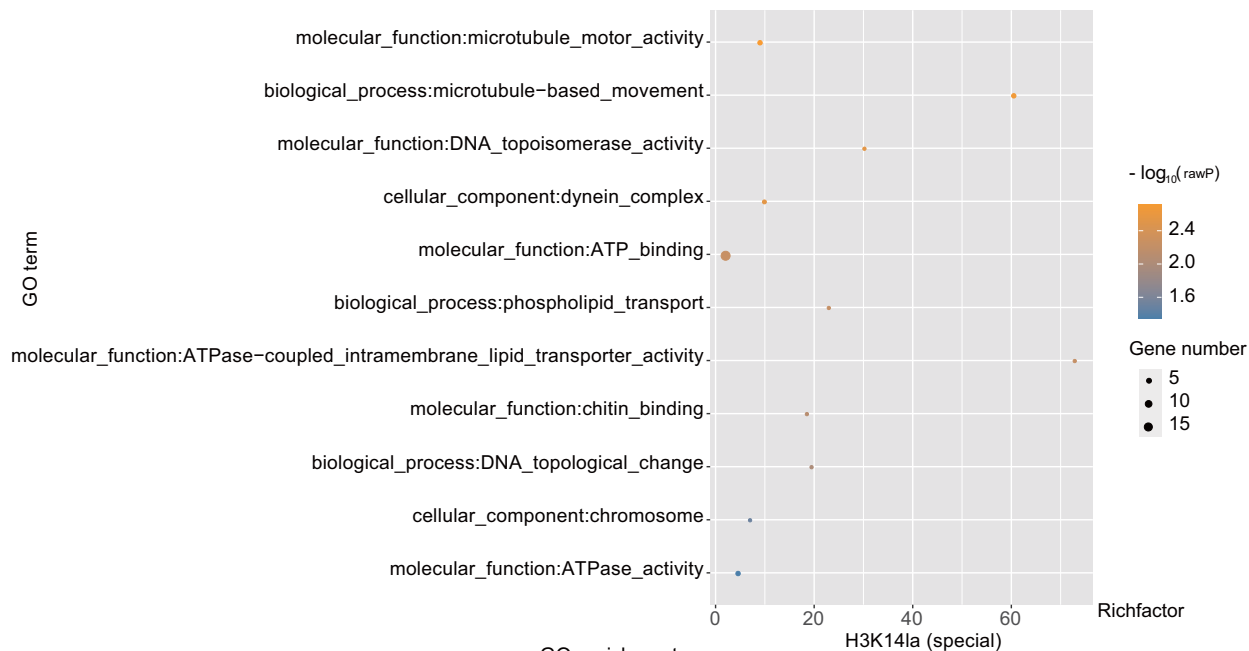

B

GO enrichment

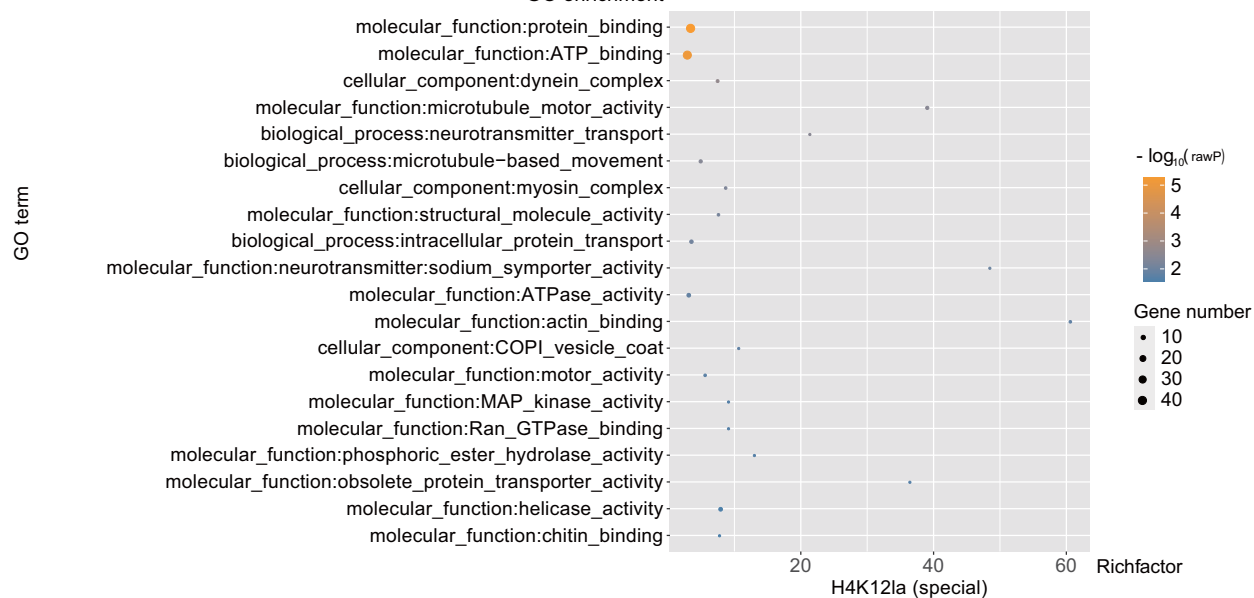

C

GO enrichment

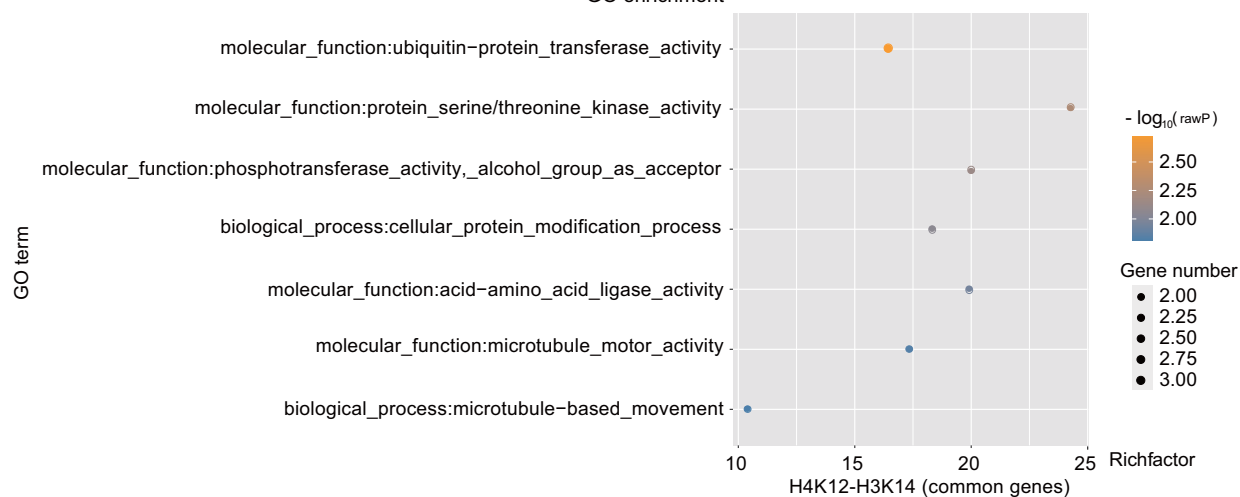

Supplement: Supplementary Figure S13 — GO enrichment analysis of genes associated with H3K14la and H4K12la A. GO enrichment analysis of unique genes related to H3K14la. B. GO enrichment analysis of unique genes related to H4K12la. C. GO Enrichment analysis of overlapped genes related to H3K14la and H4K12la. [file mmc13.pdf]

A

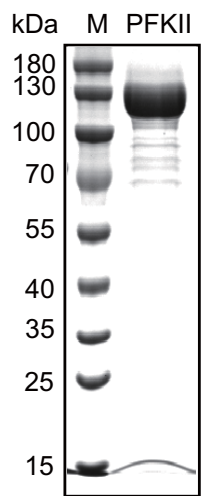

SDS-PAGE

B

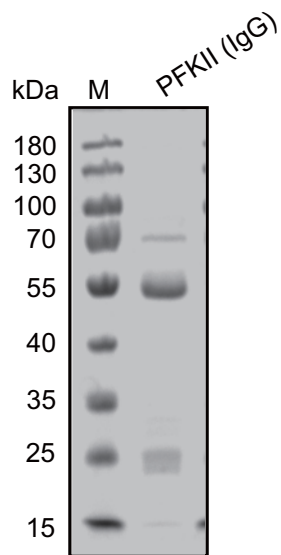

SDS-PAGE

C

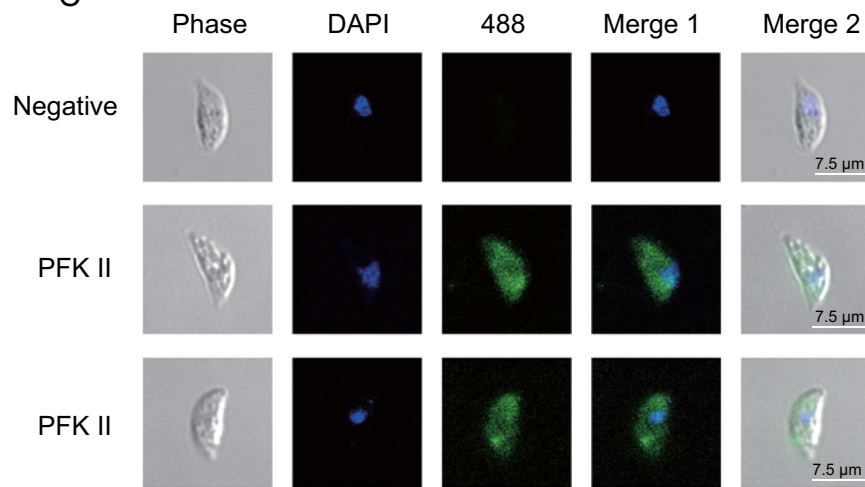

Extracellular

D

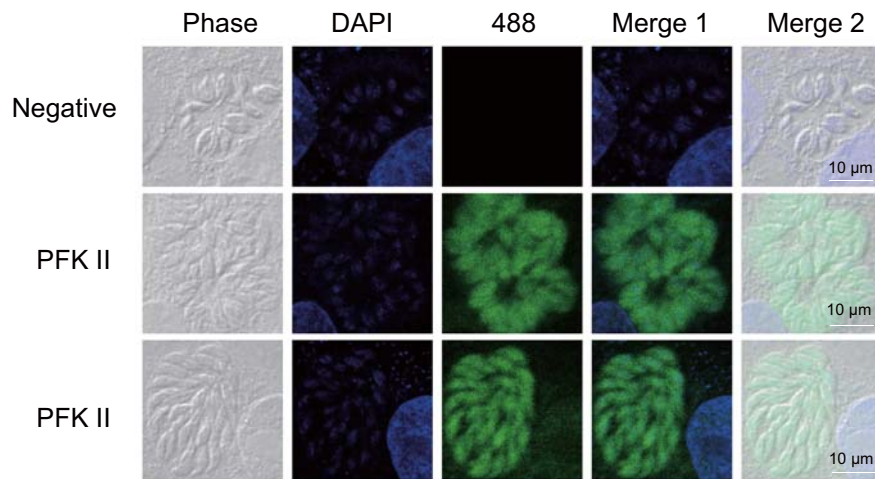

Intracellular

Supplement: Supplementary Figure S14 — Indirect IFA of TgPFKII A. Purified His-tagged TgPFKII-His was analyzed by SDS–PAGE. B. The effect of IgG purification (TgPFKII) was identified using SDS–PAGE. C. Indirect immunofluorescence of TgPFKII in the tachyzoites (green). D. Indirect immunofluorescence of TgPFKII in the parasitophorous vacuole (green). [file mmc14.pdf]

**A**

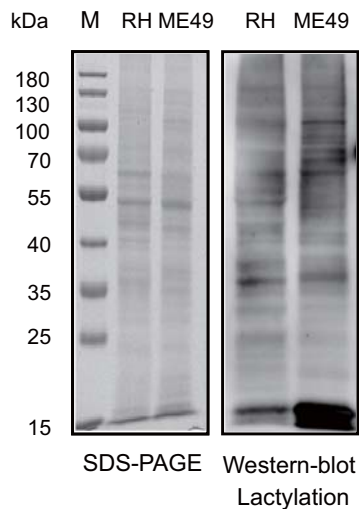

**B**

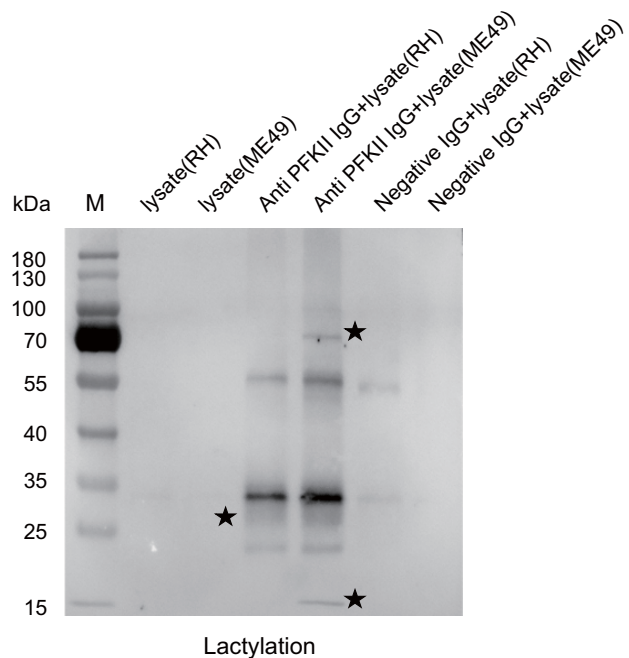

**C**

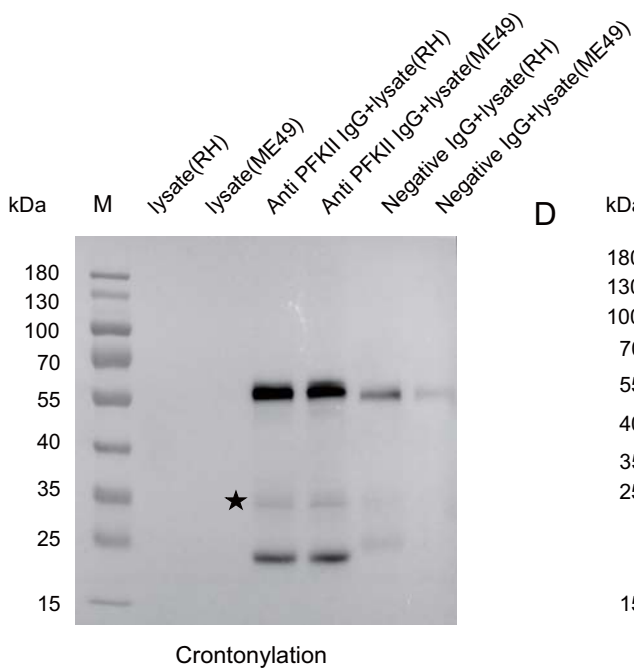

**D**

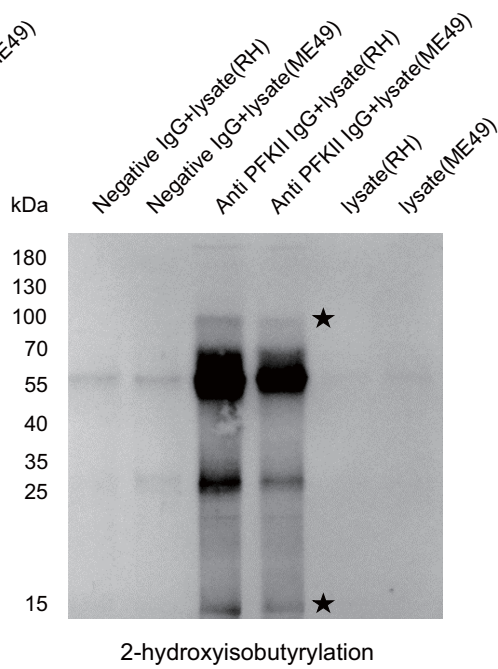

Supplement: Supplementary Figure S15 — Analysis of the difference in Kla, Kcr, and Khib levels of TgPFKII binding proteins in the T. gondii RH and ME49 strains. A. Western blotting analysis of lactylation in T. gondii. B. Analysis of the difference in the lactylation level of TgPFKII binding proteins. C. Analysis of the difference in the crotonylation level of TgPFKII binding proteins. D. Analysis of the difference in the 2-hydroxyisobutyrylation level of TgPFKII binding proteins. Kcr, lysine crotonylation; Khib, lysine 2-hydroxyisobutyrylation. [file mmc15.pdf]

A

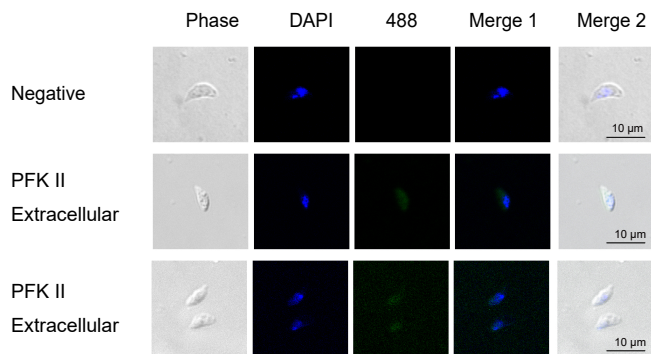*Toxoplasma*

C

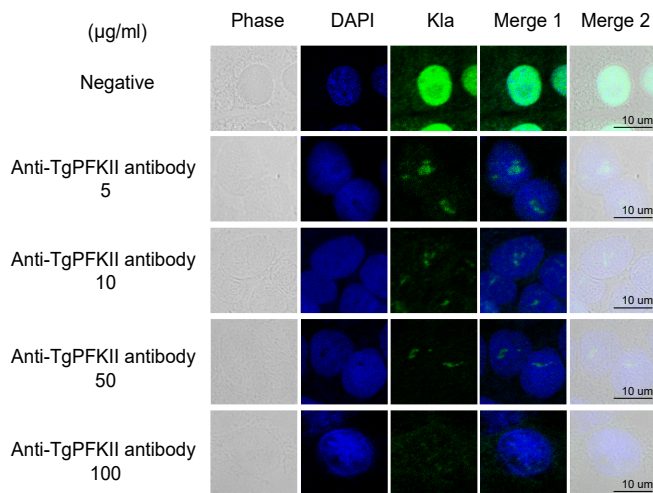

Vero cell

B

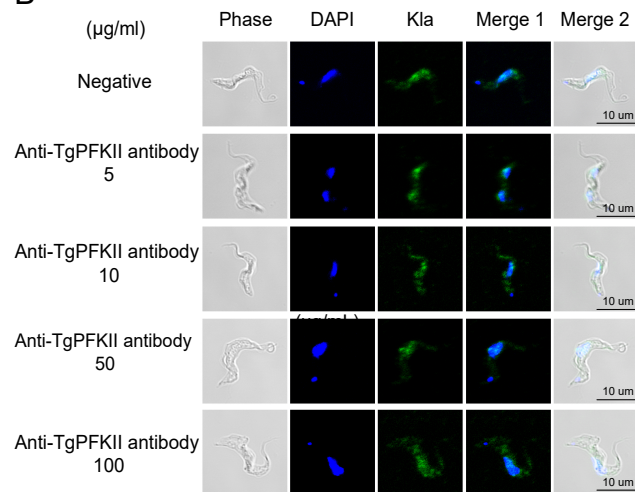*Trypanosome*

D

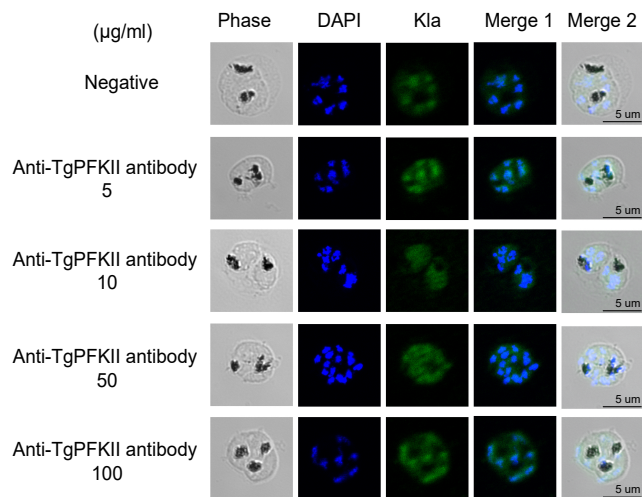*Plasmodium*

Supplement: Supplementary Figure S16 — The effect of anti-TgPFKII antibody on lactylation of Trypanosoma, Plasmodium, and Vero cells A. The permeability of anti-TgPFKII IgG to live T. gondii was verified by the indirect IFA. B. The study of the effect of anti-TgPFKII antibody on the lactylation of Trypanosome by indirect immunofluorescence. C. The study of the effect of anti-TgPFKII antibody on the lactylation of Vero cells by indirect immunofluorescence. D. The study of the effect of anti-TgPFKII antibody on the lactylation of Plasmodium by indirect immunofluorescence. [file mmc16.pdf]

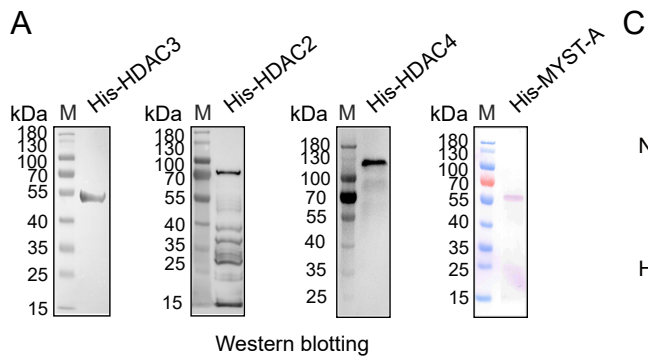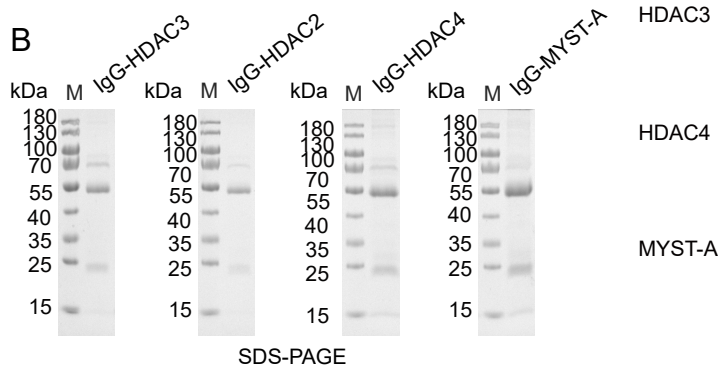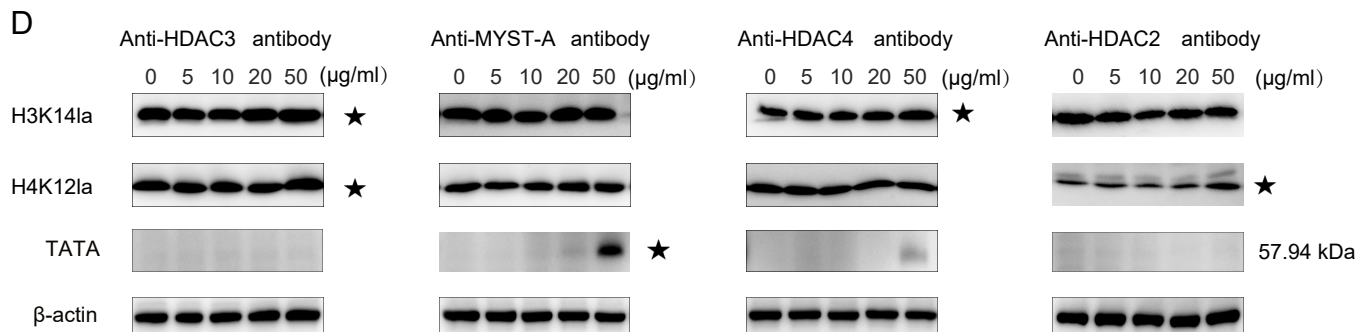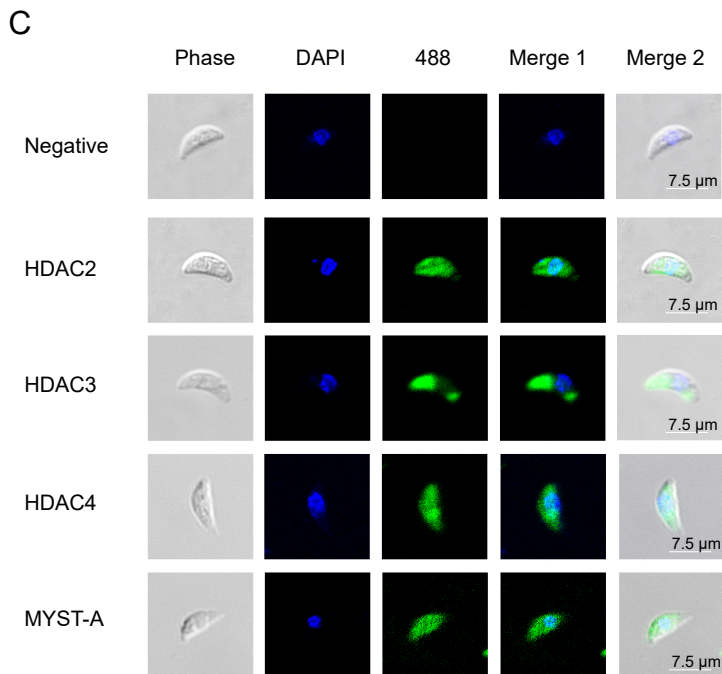

Supplement: Supplementary Figure S17 — The effect of anti-TgHDAC2, anti-TgHDAC3, anti-TgHDAC4, and anti-TgMYST-A antibodies on H3K14la, H4K12la, and TATA protein A. Western blotting analysis of recombinant proteins (TgHDAC2, TgHDAC3, TgHDAC4, and TgMYST-A). B. The effect of IgG purification was identified using SDS–PAGE. C. Indirect IFA of TgHDAC2, TgHDAC3, TgHDAC4, and TgMYST-A for the tachyzoite stage (green). Nuclei is colored in blue. D. The effect of different concentrations of antibodies (anti-TgHDAC2, TgHDAC3, TgHDAC4, and TgMYST-A) on H3K14la, H4K12la levels and the expression level of TATA protein was examined through western blotting. [file mmc17.pdf]

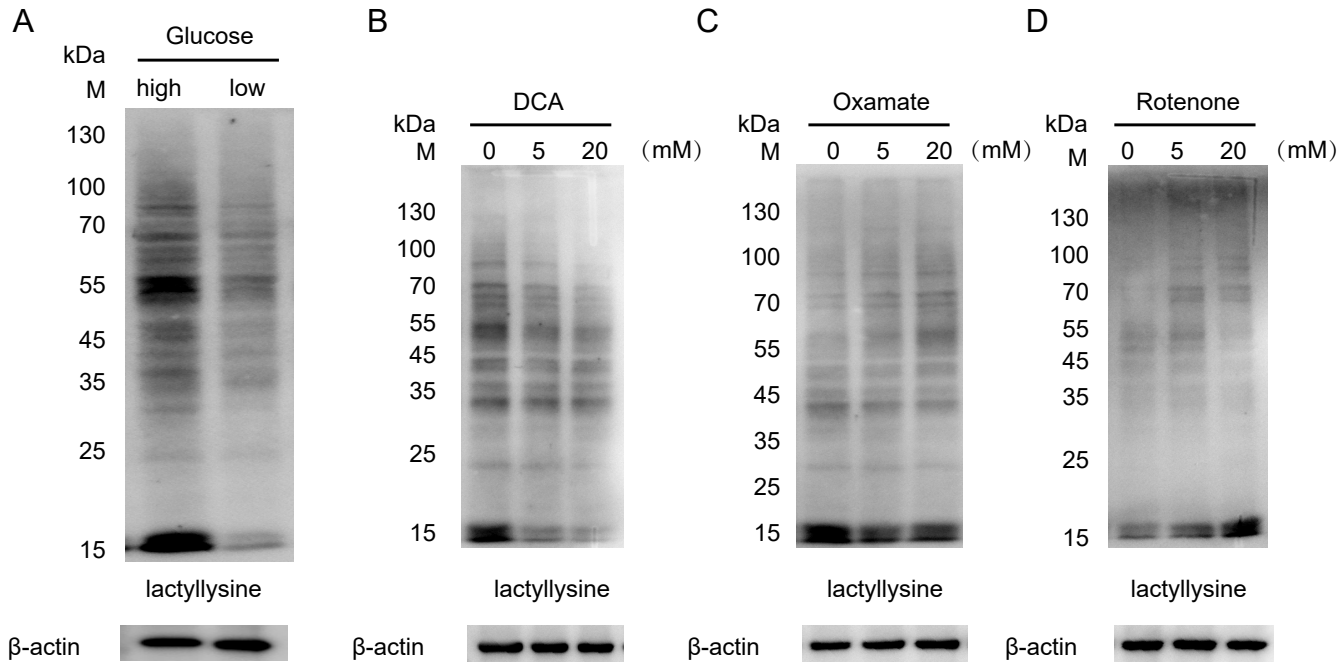

Supplement: Supplementary Figure S18 — The effect of inhibitors on lactylation A. Lactylation level of T. gondii under high and low glucose conditions was measured through Western blotting. β-Actin was used as an internal reference standard. B–D. The effect of the sodium dichloroacetate (B), oxamate (C), and rotenone (D) on the lactylation level was detected through western blotting. β-Actin was used for normalization. DCA, sodium dichloroacetate. [file mmc18.pdf]

**A**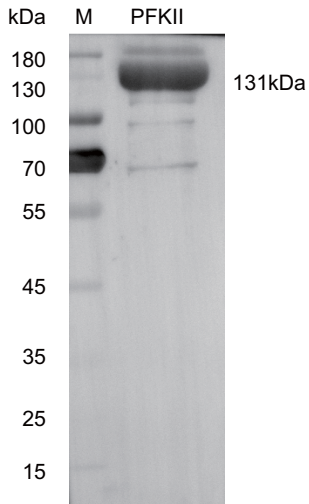

Crotonylation

**B**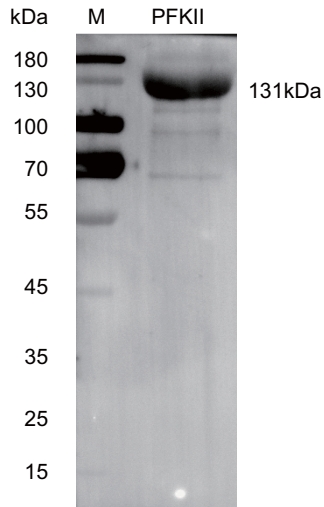

2-hydroxyisobutyrylation

Western blotting

Supplement: Supplementary Figure S19 — TgPFKII is crotonylated and 2-hydroxyisobutyrylated A. The lysine crotonylation of TgPFKII was detected through western blotting (131 kDa). B. The lysine 2-hydroxyisobutyrylation of TgPFKII was detected through western blotting (131 kDa). [file mmc19.pdf]

A

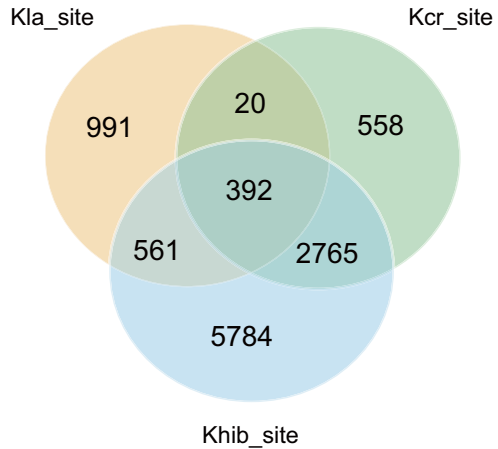

The number of site

B

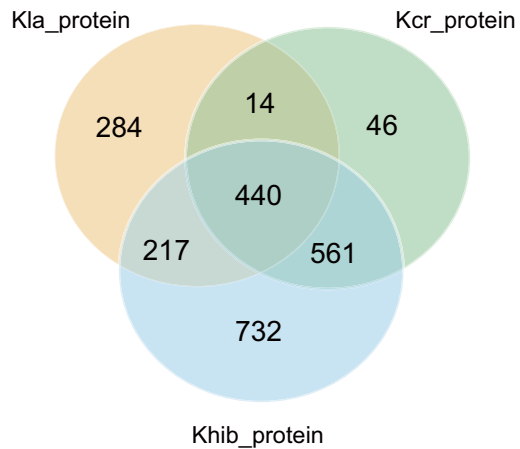

The number of protein

Supplement: Supplementary Figure S20 — Overlap analysis of Kla, Kcr, and Khib in the T. gondii RH strain A. Overlap analysis of Kla, Kcr, and Khib sites. B. Overlap analysis of Kla, Kcr, and Khib proteins. [file mmc20.pdf]

A

## Protein domain

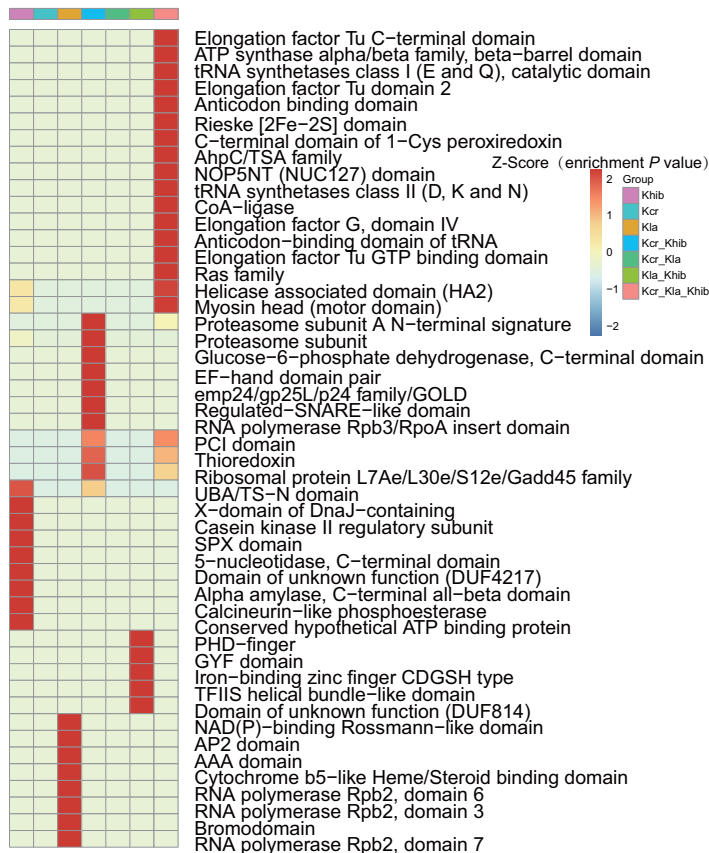

B

## KEGG pathway

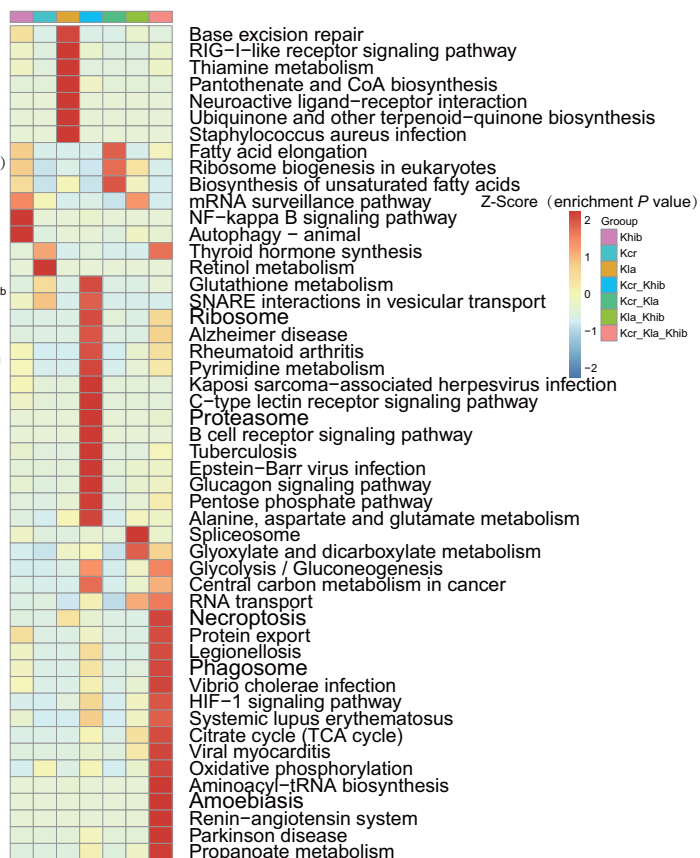

Supplement: Supplementary Figure S22 — Domain and KEGG enrichment analysis of Kla, Kcr, and Khib proteins A. Domain enrichment analysis of Kla, Kcr, and Khib proteins. B. KEGG pathway enrichment analysis of Kla, Kcr, and Khib proteins. [file mmc22.pdf]

## Glycolysis/gluconeogenesis

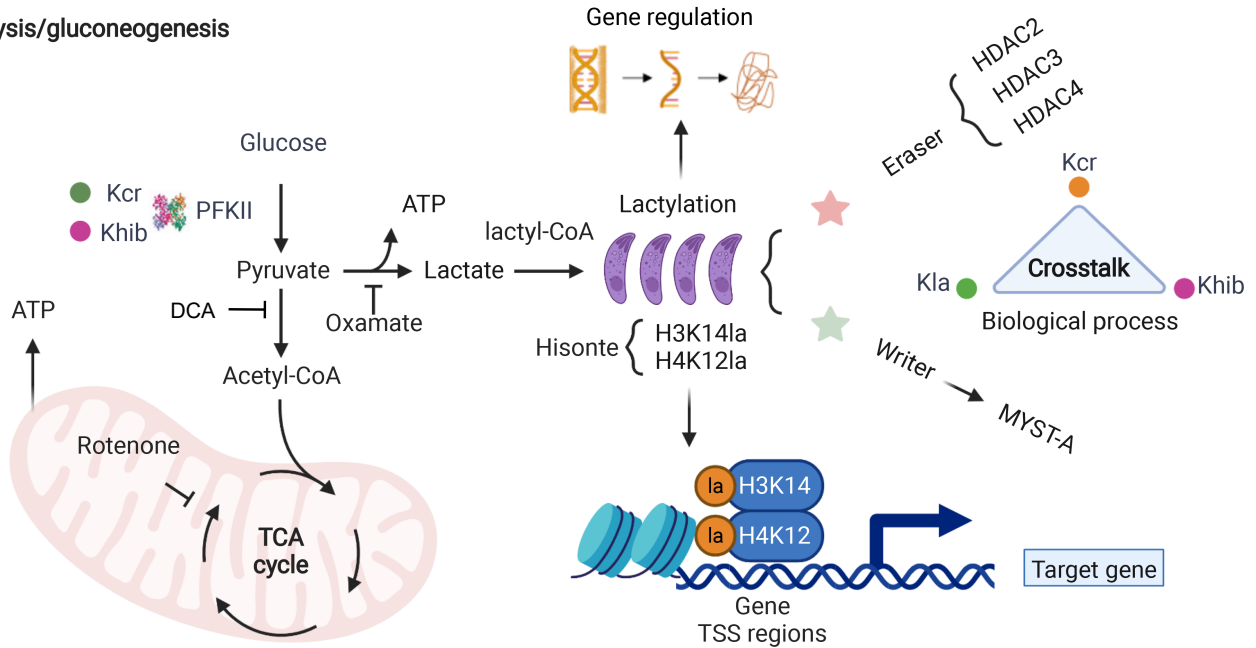

Supplement: Supplementary Figure S23 — A schematic illustration of lactylation and metabolic regulation The schematic highlights the relationship between glycolysis and protein lactylation. Figures were created using BioRender.com. ATP, adenosine triphosphate. [file mmc23.pdf]
