# Supplementary material for: Protein Lactylation and Metabolic Regulation of the Zoonotic Parasite Toxoplasma gondii
Source: Genomics Proteomics Bioinformatics. 2022 Oct 7;21(6):1163–81. doi: 10.1016/j.gpb.2022.09.010 (PMC11082259; doi:10.1016/j.gpb.2022.09.010)
Supplement: Supplementary Figure S7 — Lactylated enzymes of glycolysis/gluconeogenesis and the citrate cycle The proteins highlighted in blue are modified by lactylation. The orange circles represent the number of lactylation sites. The detailed data are provided in Table S10. TCA, tricarboxylic acid. [file mmc7.pdf]

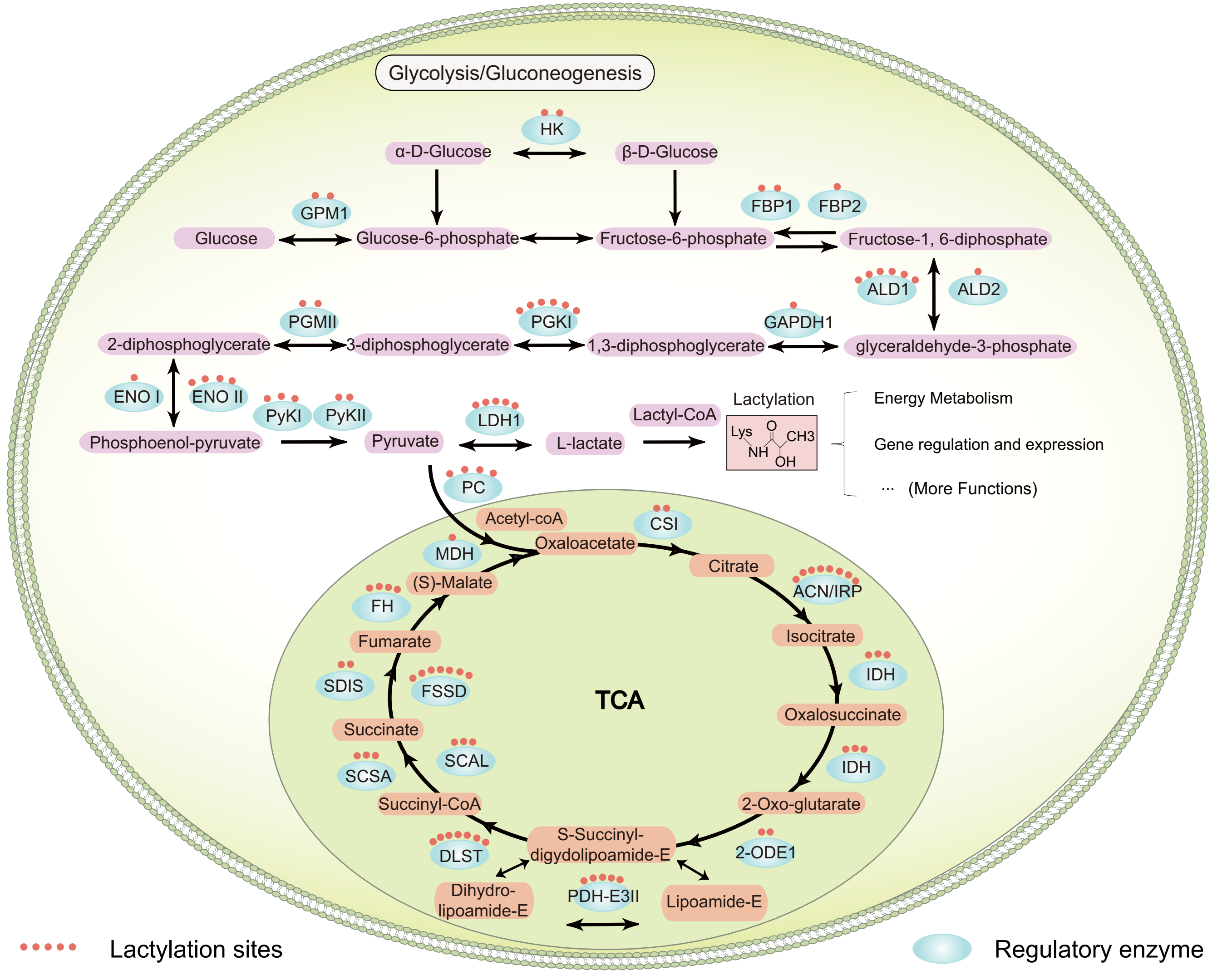
Glycolysis/Gluconeogenesis
HK
α-D-Glucose
β-D-Glucose
GPM1
FBP1
FBP2
Glucose
Glucose-6-phosphate
Fructose-6-phosphate
Fructose-1, 6-diphosphate
ALD1
ALD2
PGMII
PGKI
GAPDH1
2-diphosphoglycerate
3-diphosphoglycerate
1,3-diphosphoglycerate
glyceraldehyde-3-phosphate
ENO I
ENO II
PyKI
PyKII
LDH1
Lactyl-CoA
Lactylation
Lys
NH
O
CH3
OH
Energy Metabolism
Gene regulation and expression
··· (More Functions)
Phosphoenol-pyruvate
Pyruvate
L-lactate
PC
Acetyl-coA
CSI
MDH
Oxaloacetate
Citrate
(S)-Malate
ACN/IRP
FH
Fumarate
Isocitrate
IDH
SDIS
FSSD
TCA
Oxalosuccinate
Succinate
SCAL
IDH
SCSA
Succinyl-CoA
2-Oxo-glutarate
S-Succinyl-digydolipoamide-E
DLST
2-ODE1
Dihydro-lipoamide-E
PDH-E3II
Lipoamide-E
Lactylation sites
Regulatory enzyme
