# Supplementary material for: Protein Lactylation and Metabolic Regulation of the Zoonotic Parasite Toxoplasma gondii
Source: Genomics Proteomics Bioinformatics. 2022 Oct 7;21(6):1163–81. doi: 10.1016/j.gpb.2022.09.010 (PMC11082259; doi:10.1016/j.gpb.2022.09.010)
Supplement: Supplementary Figure S8 — Interaction network of the lactylated proteins The size of the circle represents the different node degrees. Circles of different colors represent different pathways: ribosomes (red), ribosome biogenesis in eukaryotes (green), aminoacyl-tRNA biosynthesis (purple), spliceosomes (yellow), and carbon metabolism (light blue). Gray circles represent unclassified lactylated proteins. [file mmc8.pdf]

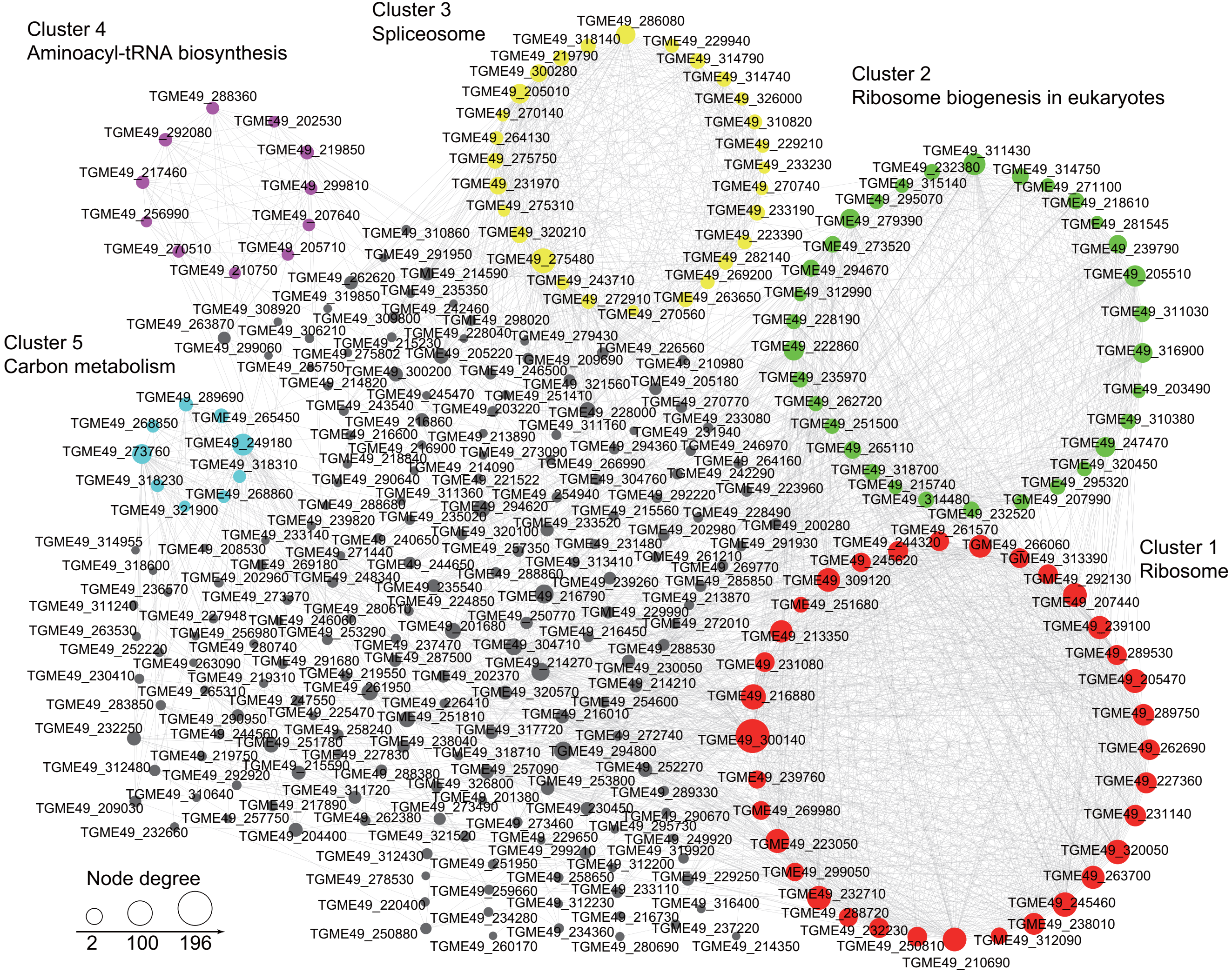
Cluster 4
Aminoacyl-tRNA biosynthesis
TGME49_288360
TGME49_202530
TGME49_292080
TGME49_219850
TGME49_217460
TGME49_299810
TGME49_256990
TGME49_207640
TGME49_270510
TGME49_205710
TGME49_210750
Cluster 3
Spliceosome
TGME49_286080
TGME49_318140
TGME49_229940
TGME49_219790
TGME49_314790
TGME49_300280
TGME49_314740
TGME49_205010
TGME49_326000
TGME49_270140
TGME49_310820
TGME49_264130
TGME49_229210
TGME49_275750
TGME49_233230
TGME49_231970
TGME49_270740
TGME49_275310
TGME49_233190
TGME49_320210
TGME49_223390
TGME49_275480
TGME49_282140
TGME49_243710
TGME49_269200
TGME49_272910
TGME49_263650
TGME49_270560
Cluster 2
Ribosome biogenesis in eukaryotes
TGME49_311430
TGME49_232380
TGME49_314750
TGME49_315140
TGME49_271100
TGME49_295070
TGME49_218610
TGME49_279390
TGME49_281545
TGME49_273520
TGME49_239790
TGME49_294670
TGME49_205510
TGME49_312990
TGME49_311030
TGME49_228190
TGME49_222860
TGME49_316900
TGME49_235970
TGME49_203490
TGME49_262720
TGME49_310380
TGME49_251500
TGME49_247470
TGME49_265110
TGME49_320450
TGME49_318700
TGME49_295320
TGME49_215740
TGME49_207990
TGME49_314480
TGME49_232520
Cluster 5
Carbon metabolism
TGME49_289690
TGME49_265450
TGME49_268850
TGME49_249180
TGME49_273760
TGME49_318310
TGME49_318230
TGME49_268860
TGME49_321900
Cluster 1
Ribosome
TGME49_261570
TGME49_244320
TGME49_266060
TGME49_313390
TGME49_245620
TGME49_292130
TGME49_309120
TGME49_207440
TGME49_251680
TGME49_239100
TGME49_213350
TGME49_289530
TGME49_231080
TGME49_205470
TGME49_216880
TGME49_289750
TGME49_300140
TGME49_262690
TGME49_239760
TGME49_227360
TGME49_269980
TGME49_231140
TGME49_223050
TGME49_320050
TGME49_299050
TGME49_263700
TGME49_232710
TGME49_245460
TGME49_288720
TGME49_238010
TGME49_232230
TGME49_250810
TGME49_312090
TGME49_210690
Node degree
2
100
196
