# Supplementary material for: Protein Lactylation and Metabolic Regulation of the Zoonotic Parasite Toxoplasma gondii
Source: Genomics Proteomics Bioinformatics. 2022 Oct 7;21(6):1163–81. doi: 10.1016/j.gpb.2022.09.010 (PMC11082259; doi:10.1016/j.gpb.2022.09.010)
Supplement: Supplementary Table S9 — Lactylated proteins related to spliceosome [file mmc32.docx]

**Table S9 Lactylated proteins related to spliceosome**

| **Name** | **Gene** | **Protein description** | **Sites (K)** |
| --- | --- | --- | --- |
| Sm | TGME49_314790 | small nuclear ribonucleoprotein G, putative | 7 |
|  | TGME49_275750 | small nuclear ribonucleoprotein E, putative | 9 |
|  | TGME49_300280 | LSM domain-containing protein | 55 |
| U1-70k | TGME49_205180 | RNA recognition motif-containing protein | 82 |
| U1A | TGME49_309800 | RNA recognition motif-containing protein | 275 |
| S164 | TGME49_270770 | PWI domain-containing protein | 614, 660 |
| U2A’ | TGME49_229210 | small nuclear ribonucleoprotein polypeptide A', putative | 178 |
| U2B’’ | TGME49_209690 | small nuclear ribonucleoprotein | 147 |
| SF3a | TGME49_228000 | splicing factor 3A subunit 2, putative | 93, 104 |
| SF3b | TGME49_314740 | PSP protein | 749 |
|  | TGME49_205010 | U2 small nuclear ribonucleoprotein family protein, putative | 281 |
|  | TGME49_203220 | DEAD/DEAH box helicase domain-containing protein | 838 |
| U2AF | TGME49_291950 | RNA recognition motif-containing protein | 20, 31, 566 |
|  | TGME49_308920 | splicing factor U2AF protein | 68, 109 |
|  | TGME49_319850 | splicing factor U2AF family SnRNP auxilary factor large subunit, RRM domain-containing protein | 178, 223 |
| SPF45 | TGME49_214820 | G-patch domain-containing protein | 127 |
| CHERP | TGME49_321560 | zinc knuckle domain-containing protein | 534 |
| Prp43 | TGME49_233520 | ATP-dependent RNA helicase | 7, 446 |
| Prp3 | TGME49_219790 | pre-mRNA processing factor PRP3 | 244 |
| Prp4 | TGME49_243540 | WD domain, G-beta repeat-containing protein | 80 |
| Sad1 | TGME49_294360 | ubiquitin specific protease 39 isoform 2, putative | 50 |
| SSu66 | TGME49_318140 | SART-1 family protein | 436, 474 |
| Snu23 | TGME49_275310 | U1-type domain-containing protein(predict) | 321, 349 |
| Prp38 | TGME49_285230 | PRP38 family protein | 493, 576 |
| Prp18 | TGME49_320210 | WD domain, G-beta repeat domain containing protein | 33, 290 |
| CDC5 | TGME49_275480 | Myb family DNA-binding domain-containing protein | 120 |
| AD002 | TGME49_270740 | Cwf15/Cwc15 cell cycle control protein | 28, 44 |
| HSP73 | TGME49_273760 | heat shock protein HSP70 | 56, 71, 77, 112, 138, 188, 253, 321, 324, 425, 499, 509, 526, 599 |
| Skip | TGME49_273760 | SKIP/SNW domain-containing protein | 288, 310, 307 |
| Syf | TGME49_269200 | crooked neck family 1 protein isoform 2, putative | 34, 36, 84 |
| PPIL1 | TGME49_270560 | peptidyl-prolyl cis-trans isomerase family 1 | 12 |
| CCDC12 | TGME49_279430 | cwf18 pre-mRNA splicing factor protein | 115 |
| RBM22 | TGME49_326000 | RNA binding protein, putative | 6 |
|  | TGME49_325900 | zinc finger (CCCH type) motif-containing protein | 6, 138 |
| Y14 | TGME49_233230 | RNA-binding protein 8A family protein | 64 |
| THOC | TGME49_254500 | WD domain, G-beta repeat protein(predict) | 290 |
| SR | TGME49_211420 | RNA recognition motif-containing protein | 62, 89 |
|  | TGME49_304760 | RNA recognition motif-containing protein | 428, 519, 949 |
| Snu114 | TGME49_286080 | elongation factor 2 family protein | 70, 396 |
| Bn2 | TGME49_223390 | activating signal cointegrator 1 complex subunit 3 family 1 ASCC3L1 | 49, 581 |
| Prp6 | TGME49_205220 | U5 snRNP-associated subunit, putative | 1043 |
| Prp8 | TGME49_231970 | pre-mRNA processing splicing factor PRP8 | 223, 216, 166 |
| Prp8BP | TGME49_310860 | U5 snRNP-specific protein | 320 |
| Prp28 | TGME49_298020 | DEAD-family helicase | 97, 475 |
| DIB1 | TGME49_270140 | splicing factor DIM1, putative | 134, 139 |
| Prp5 | TGME49_221660 | DEAD/DEAH box helicase domain-containing protein | 233, 292, 231, 494 |
| Prp2 | TGME49_263650 | pre-mRNA-processing protein 8, putative | 303 |
| Slu7 | TGME49_310820 | SLU7 splicing factor, putative | 301 |
| Prp43 | TGME49_233520 | ATP-dependent RNA helicase | 7446 |
