# Supplementary material for: Protein Lactylation and Metabolic Regulation of the Zoonotic Parasite Toxoplasma gondii
Source: Genomics Proteomics Bioinformatics. 2022 Oct 7;21(6):1163–81. doi: 10.1016/j.gpb.2022.09.010 (PMC11082259; doi:10.1016/j.gpb.2022.09.010)
Supplement: Supplementary Table S10 — Modified enzymes involved in energy metabolism [file mmc33.docx]

**Table S10 Modified enzymes involved in energy metabolism**

|  | **Gene** | **Protein description** | **Sites (K)** |
| --- | --- | --- | --- |
| **Glycolysis/ Gluconeogenesis** | TGME49_285980 | glucosephosphate-mutase GPM1 | 193, 461 |
|  | TGME49_205380 | fructose-bisphospatase I | 92, 246 |
|  | TGME49_247510 | fructose-bisphospatase II | 249 |
|  | TGME49_236040 | fructose-1,6-bisphosphate aldolase(ALD1) | 177, 180, 429, 206, 393, 111 |
|  | TGME49_321900 | fructose-1,6-bisphosphate aldolase(ALD2) | 220 |
|  | TGME49_289690 | glyceraldehyde-3-phosphate dehydrogenase GAPDH1 | 366, 214, 405, 255, 370, 274 |
|  | TGME49_318230 | phosphoglycerate kinase PGKI | 139, 141, 31, 206, 92, 103 |
|  | TGME49_297060 | phosphoglycerate mutase PGMII | 337, 328 |
|  | TGME49_268850 | enolase 2 | 162, 103, 112, 405 |
|  | TGME49_268860 | enolase 1 | 81 |
|  | TGME49_256760 | pyruvate kinase PyKI | 109, 136, 4 |
|  | TGME49_299070 | pyruvate kinase PyKII | 336, 328 |
|  | TGME49_232350 | lactate dehydrogenase LDH1 | 211, 43, 322, 218, 94 |
|  | TGME49_265450 | hexokinase | 103, 446 |
| **Citrate cycle** | TGME49_268890 | citrate synthase I | 410, 423 |
|  | TGME49_226730 | aconitate hydratase ACN/IRP | 785, 296, 223, 895, 1049, 788, 863, 300 |
|  | TGME49_313140 | isocitrate dehydrogenase | 227, 265, 256 |
|  | TGME49_244200 | 2-oxoglutarate dehydrogenase e1 component, mitochondrial precursor, putative (2-ODE1) | 704, 1112 |
|  | TGME49_318430 | malate dehydrogenase MDH | 509 |
|  | TGME49_219550 | dihydrolipoyllysine-residue succinyltransferase component of oxoglutarate dehydrogenase (DLST) | 219, 226, 198, 205, 193, 370, 225 |
|  | TGME49_290600 | succinyl-CoA-synthetase alpha SCSA | 74, 166, 94 |
|  | TGME49_309752 | succinate-Coenzyme A ligase, beta subunit, putative (SCAL) | 172, 315, 279 |
|  | TGME49_215280 | succinate dehydrogenase [ubiquinone] iron-sulfur protein (SDIS) | 205, 330 |
|  | TGME49_215590 | flavoprotein subunit of succinate dehydrogenase (FSSD) | 194, 351, 497, 623, 160, 572, 666 |
|  | TGME49_206470 | pyruvate dehydrogenase complex subunit PDH-E3II | 420, 514, 276, 176, 455 |
|  | TGME49_284190 | pyruvate carboxylase | 1335, 1339, 1120, 1309 |
