# Supplementary material for: Protein Lactylation and Metabolic Regulation of the Zoonotic Parasite Toxoplasma gondii
Source: Genomics Proteomics Bioinformatics. 2022 Oct 7;21(6):1163–81. doi: 10.1016/j.gpb.2022.09.010 (PMC11082259; doi:10.1016/j.gpb.2022.09.010)
Supplement: Supplementary Table S11 — Lactylated proteins involved in oxidative phosphorylation [file mmc34.docx]

**Table S11 Lactylated proteins involved in oxidative phosphorylation**

| **Gene** | **Protein description** | **Sites (K)** |
| --- | --- | --- |
| TGME49_215590 | flavoprotein subunit of succinate dehydrogenase | 194, 351, 497, 623, 160, 572, 666 |
| TGME49_215280 | succinate dehydrogenase [ubiquinone] iron-sulfur protein | 205, 330 |
| TGME49_320220 | ubiquinol cytochrome c oxidoreductase, putative | 253 |
| TGME49_283830 | type I inorganic pyrophosphatase PPase | 267 |
| TGME49_261950 | ATP synthase beta subunit ATP-B | 69, 182, 551, 286 |
| TGME49_208590 | vacuolar ATP synthase subunit 54kD, putative | 66 |
| TGME49_284540 | ATP synthase F1, delta subunit protein | 194 |
| TGME49_315620 | vacuolar ATP synthase subunit C, putative | 170 |
| TGME49_219800 | vacuolar ATP synthase subunit b, putative | 7 |
| TGME49_305290 | vacuolar atp synthase subunit e, putative | 262 |
| TGME49_256970- | vacuolar ATP synthase subunit A, putative | 128 |
| TGME49_231910 | ATP synthase F1 gamma subunit | 100 |
| TGME49_204400 | ATPase synthase subunit alpha, putative | 71, 175, 436, 511 |
| TGME49_240550 | copper chaperone COX17-1, putative | 33 |
