# Supplementary material for: Protein Lactylation and Metabolic Regulation of the Zoonotic Parasite Toxoplasma gondii
Source: Genomics Proteomics Bioinformatics. 2022 Oct 7;21(6):1163–81. doi: 10.1016/j.gpb.2022.09.010 (PMC11082259; doi:10.1016/j.gpb.2022.09.010)
Supplement: Supplementary Table S12 — Lactylation of invasion-associated proteins [file mmc35.docx]

**Table S12 Lactylation of invasion-associated proteins**

| **Gene** | **Protein description** | **Sites (K)** |
| --- | --- | --- |
| TGME49_209030 | actin ACT1 | 40, 114, 327, 316, 329, 51 |
| TGME49_297470 | myosin light chain 2, putative | 211, 279, 210, 248 |
| TGME49_235470 | myosin A | 386,745, 426, 17,567, 11 |
| TGME49_255190 | myosin C | 27 |
| TGME49_263180 | myosin D | 211, 10, 725, 517, 628, 567, 368 |
| TGME49_220400 | actin depolymerizing factor ADF | 91 |
| TGME49_278870 | myosin F | 1191 |
| TGME49_291020 | myosin head (motor domain) domain-containing protein | 1093 |
| TGME49_243730 | rhoptry protein ROP9 | 144, 79, 138, 347 |
| TGME49_205250 | rhoptry protein ROP18 | 202 |
| TGME49_297960 | rhoptry neck protein RON6 | 1170 |
| TGME49_315210 | rhoptry protein, putative | 954,956 |
| TGME49_291890 | microneme protein MIC1 | 157 |
| TGME49_201780 | microneme protein MIC2 | 630 |
| TGME49_288650 | dense granule protein GRA12 | 74 |
| TGME49_301440 | calcium-dependent protein kinase CDPK1 | 59, 80, 93, 50, 341 |
| TGME49_206590 | calcium-dependent protein kinase CDPK2A | 165, 777, 729 |
| TGME49_217600 | calcium-dependent protein kinase CDPK9 | 5 |
