# Supplementary material for: Protein Lactylation and Metabolic Regulation of the Zoonotic Parasite Toxoplasma gondii
Source: Genomics Proteomics Bioinformatics. 2022 Oct 7;21(6):1163–81. doi: 10.1016/j.gpb.2022.09.010 (PMC11082259; doi:10.1016/j.gpb.2022.09.010)
Supplement: Supplementary Table S24 — Proteomic studies of PTMs in Toxoplasma gondii [file mmc47.docx]

**Table S24 Proteomic studies of PTMs in *Toxoplasma gondii***

| **Strain** | **Parasite stage** | **PTM types** | **Proteins** | **PTM sites** | **Proteome**  **coverage (%)** | **Function** | **References** |
| --- | --- | --- | --- | --- | --- | --- | --- |
| RH | Intracellular | Lysine acetylation | 274 | 411 | 3.3% | transcription, translation, metabolism, and stress responses | (Jeffers and  Sullivan et al., 2012) |
| RH | Extracellular | Lysine acetylation | 386 | 571 | 4.6% | metabolism, translation, and chromatin biology | (Xue et al., 2013) |
| RH | Intra-and extracellular | Arginine  Methylation | 370 | 618 | 4.5% | transcriptional regulation and splicing biology | (Yakubu, et al., 2017) |
| GT1 | Extracellular | O-GlcNAcylation |  |  |  | cell cycle, intracellular transport of proteins, protein turnover, | (Perez-Cervera,Y et al., 2011) |
| RH | Intracellular | Cysteine palmitoylation  myristoylation, prenylation | 401 |  | 4.8% | metabolic processes, transcription and translation, gliding and host-cell invasion | (Caballero et al., 2016) |
| RH | Intracellular | Cysteine palmitoylation | 282 |  | 3.4% | invasion, motility, and cell morphology | (Foe et al., 2015) |
| RH |  | Cysteine palmitoylation | 112 |  |  | host cell invasion, parasite motility and organelle biogenesis | (Frenal et al., 2013) |
| RH | Intra-and extracellular | Ubiquitination | 454 | 800 | 5.4% | cell division and cell cycle | (Silmon de Monerri et al., 2015) |
| RH | Extracellular | SUMO | 120 |  | 1.4% | host cell invasion and cyst genesis | (Braun et al., 2009) |
| RH | Extracellular  tachyzoites | Lysine succinylation | 147 | 425 | 1.8% | metabolism, epigenetic gene regulation | (Li et al., 2014) |
| RH | Intracellular | Phosphorylation | 892 | 1,619 | 10.6% | host cell signaling activation/deactivation | (He et al., 2017) |
| RH | Intracellular  Intracellular (Purified) | Phosphorylation | 2793  3506 | 12,793  24,298 | 33.2%  41.64% | host-pathogen interaction | (Treeck et al., 2011) |
| RH | Extracellular | Lysine crotonylation | 1061 | 3735 | 12.6% | ribosome, proteasome, pentose phosphate pathway, microbial metabolism in diverse environments, glycolysis/ gluconeogenesis, citrate cycle (TCA cycle), Aminoacyl−tRNA biosynthesis, carbon metabolism, Biosynthesis of amino acids, peroxisome, carbon fixation in photosynthetic organisms, gap junction, 2-oxocarboxylic acid metabolism, glutathione metabolism, MAPK signaling pathway, ferroptosis | (Yin et al., 2019) |
| RH | Extracellular | Lysine 2-hydroxyisobutyrylation | 1950 | 9502 | 23.2% | ribosome, proteasome, glycolysis / gluconeogenesis, Citrate cycle (TCA cycle), Carbon metabolism, Biosynthesis of amino acids, Carbon fixation in photosynthetic organisms, spliceosome, peroxisome | (Yin et al., 2019) |
| RH | Extracellular | Lysine lactylation | 955 | 1964 | 11.5% | Spliceosome, Citrate cycle (TCA cycle), Aminoacyl-tRNA biosynthesis, RNA transport, Glycolysis / Gluconeogenesis, Glyoxylate and dicarboxylate metabolism, Oxidative phosphorylation, HIF-1 signaling pathway |  |
| RH | Extracellular | N-myristoylation | 157 |  | 1.9% | metabolic functions | (Alonso et al., 2019) |
| RH | Extracellular | Malonylation | 326 |  | 506 | aminoacyl-tRNA biosynthesis, energy metabolism and fatty acid biosynthesis | (Nie et al., 2020) |
| RH | Extracellular | Lysine acetylation | 458 |  | 5.5% | biosynthesis of secondary metabolites,  biosynthesis of antibiotics, microbial metabolism in diverse environments, pyruvate metabolism | (Wang et al., 2019) |
| ME49 | Extracellular | Lysine 2-hydroxyisobutyrylation | 1720 | 8092 | 20.5% | Ribosome, Proteasome, Glycolysis / Gluconeogenesis, Citrate cycle (TCA cycle), Carbon metabolism, Aminoacyl−tRNA biosynthesis, Carbon fixation in photosynthetic organisms, Alanine, aspartate and glutamate metabolism | (Yin et al., 2019) |
| ME49 | Extracellular | Lysine crotonylation | 984 | 3396 | 10.7% | ribosome, proteasome, pentose phosphate pathway, microbial metabolism in diverse environments, citrate cycle (TCA cycle), carbon metabolism, aminoacyl−tRNA biosynthesis, RNA transport,  glycolysis/gluconeogenesis, oxidative phosphorylation | (Yin et al., 2019) |
