# Supplementary material for: Protein Lactylation and Metabolic Regulation of the Zoonotic Parasite Toxoplasma gondii
Source: Genomics Proteomics Bioinformatics. 2022 Oct 7;21(6):1163–81. doi: 10.1016/j.gpb.2022.09.010 (PMC11082259; doi:10.1016/j.gpb.2022.09.010)
Supplement: Supplementary Table S31 — Primers used in this study [file mmc54.docx]

**Table S31 Primers used in this study**

| **Primers** | **Sequence (5'-3')** | **Used for** |
| --- | --- | --- |
| *PFKII*_primer F | GTGGACAGCAAATGGGTCGCGGATCCATGACCTTCCTGAGCTTCTTTAAATG | To amplify coding sequence of PFKII (PET-28a) |
| *PFKII*_primer R | CAGTGGTGGTGGTGGTGGTGCTCGAGTTACTGGTCACGGCACGCCG |  |
| *PFKII_*primer F | AATCGGATCTGGTTCCGCGTGGATCCATGACCTTCCTGAGCTTCTTTAAATGTGCTAAA | To amplify coding sequence of PFKII (pGEX-4T-1) |
| *PFKII_*primer R | AGTCAGTCACGATGCGGCCGCTCGAGTTACTGGTCACGGCACGCCGCTGCGCCACAAGC |  |
| *HDAC3*_primer F | GCGGATCCATGGCGCTCAGTGCGCTGC | To amplify coding sequence of HDAC3 (PET-28a and pGEX-4T-1) |
| *HDAC3*_primer R | TGCTCGAGCTAGATCGGAACCTTTTGGTCTCTG |  |
| *HDAC2_*primer F | CATATGGGACGCAGAAAGTCTGTTCTG | To amplify coding sequence of HDAC2 (PET-28a) |
| *HDAC2*_primer R | CTCGAGTTAGCCGCGGTTGCGGTTAGAGATC |  |
| *HDAC2*_primer F | GAATTCATGGGACGCAGAAAGTCTGTTC | To amplify coding sequence of HDAC2 (pGEX-4T-1) |
| *HDAC2*_primer R | CTCGAGTTAGCCGCGGTTGCGGTTAGAGATC |  |
| *HDAC4*_primer F | CATATGTCTCTCCACGCGGGACACGCGCTC | To amplify coding sequence of HDAC4 (PET-28a) |
| *HDAC4*_primer R | CTCGAGTTAGCCGACCTCGTTGAGCTGG |  |
| *HDAC4*_primer F | CATATGGGACGCAGAAAGTCTGTTCTG | To amplify coding sequence of HDAC4 (pGEX-4T-1) |
| *HDAC4*_primer R | CTCGAGTTAGCCGCGGTTGCGGTTAGAGATC |  |
| *MYST-A*_primer F | CGGGATCCATGAAGAGAGTCTCGGGAGCG | To amplify coding sequence of MYST-A (PET-28a and pGEX-4T-1) |
| *MYST-A*_primer R | TAAAGCGGCCGCTTAGGCTTGAGGACTGTACTCAAAAGG |  |
