# Supplementary material for: Protein Lactylation and Metabolic Regulation of the Zoonotic Parasite Toxoplasma gondii
Source: Genomics Proteomics Bioinformatics. 2022 Oct 7;21(6):1163–81. doi: 10.1016/j.gpb.2022.09.010 (PMC11082259; doi:10.1016/j.gpb.2022.09.010)
Supplement: Supplementary Figure S21 — GO enrichment analysis of Kla, Kcr, and Khib in the T. gondii RH strain A. GO functional enrichment analysis (cellular component) of Kla, Kcr, and Khib proteins. B. GO functional enrichment analysis (molecular function) of Kla, Kcr, and Khib proteins. C. GO functional enrichment analysis (biological process) of Kla, Kcr, and Khib proteins. [file mmc21.pdf]

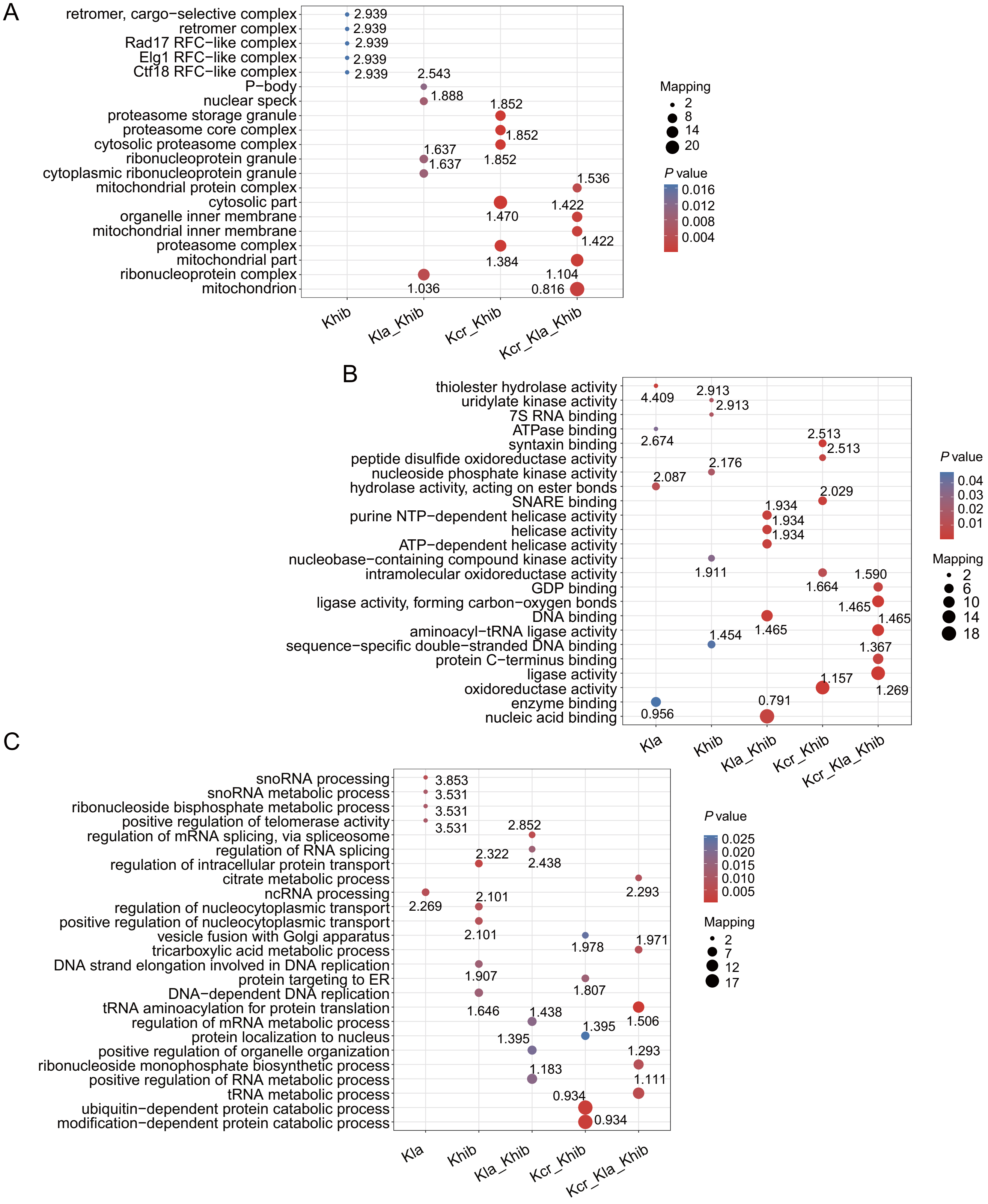
A
retromer, cargo−selective complex
retromer complex
Rad17 RFC−like complex
Elg1 RFC−like complex
Ctf18 RFC−like complex
P−body
nuclear speck
proteasome storage granule
proteasome core complex
cytosolic proteasome complex
ribonucleoprotein granule
cytoplasmic ribonucleoprotein granule
mitochondrial protein complex
cytosolic part
organelle inner membrane
mitochondrial inner membrane
proteasome complex
mitochondrial part
ribonucleoprotein complex
mitochondrion
Khib
Kla_Khib
Kcr_Khib
Kcr_Kla_Khib
Mapping
P value
B
thiolester hydrolase activity
uridylate kinase activity
7S RNA binding
ATPase binding
syntaxin binding
peptide disulfide oxidoreductase activity
nucleoside phosphate kinase activity
hydrolase activity, acting on ester bonds
SNARE binding
purine NTP−dependent helicase activity
helicase activity
ATP−dependent helicase activity
nucleobase−containing compound kinase activity
intramolecular oxidoreductase activity
GDP binding
ligase activity, forming carbon−oxygen bonds
DNA binding
aminoacyl−tRNA ligase activity
sequence−specific double−stranded DNA binding
protein C−terminus binding
ligase activity
oxidoreductase activity
enzyme binding
nucleic acid binding
Kla
Khib
Kla_Khib
Kcr_Khib
Kcr_Kla_Khib
P value
Mapping
C
snoRNA processing
snoRNA metabolic process
ribonucleoside bisphosphate metabolic process
positive regulation of telomerase activity
regulation of mRNA splicing, via spliceosome
regulation of RNA splicing
regulation of intracellular protein transport
citrate metabolic process
ncRNA processing
regulation of nucleocytoplasmic transport
positive regulation of nucleocytoplasmic transport
vesicle fusion with Golgi apparatus
tricarboxylic acid metabolic process
DNA strand elongation involved in DNA replication
protein targeting to ER
DNA−dependent DNA replication
tRNA aminoacylation for protein translation
regulation of mRNA metabolic process
protein localization to nucleus
positive regulation of organelle organization
ribonucleoside monophosphate biosynthetic process
positive regulation of RNA metabolic process
tRNA metabolic process
ubiquitin−dependent protein catabolic process
modification−dependent protein catabolic process
Kla
Khib
Kla_Khib
Kcr_Khib
Kcr_Kla_Khib
P value
Mapping
